# Resource Availability Alters Biodiversity Effects in Experimental Grass-Forb Mixtures

**DOI:** 10.1371/journal.pone.0158110

**Published:** 2016-06-24

**Authors:** Alrun Siebenkäs, Jens Schumacher, Christiane Roscher

**Affiliations:** 1 UFZ, Helmholtz Centre for Environmental Research, Department of Community Ecology, Theodor-Lieser-Strasse 4, 06120, Halle, Germany; 2 Institute of Mathematics, Stochastics, Friedrich Schiller University Jena, Ernst-Abbe-Platz 2, 07743, Jena, Germany; 3 UFZ, Helmholtz Centre for Environmental Research, Physiological Diversity, Permoserstrasse 15, 04318, Leipzig, Germany; 4 German Centre for Integrative Biodiversity Research (iDiv) Halle-Jena-Leipzig, Deutscher Platz 5e, 04103, Leipzig, Germany; Estacion Experimental de Zonas Aridas - CSIC, SPAIN

## Abstract

Numerous experiments, mostly performed in particular environments, have shown positive diversity-productivity relationships. Although the complementary use of resources is discussed as an important mechanism explaining diversity effects, less is known about how resource availability controls the strength of diversity effects and how this response depends on the functional composition of plant communities. We studied aboveground biomass production in experimental monocultures, two- and four-species mixtures assembled from two independent pools of four perennial grassland species, each representing two functional groups (grasses, forbs) and two growth statures (small, tall), and exposed to different combinations of light and nutrient availability. On average, shade led to a decrease in aboveground biomass production of 24% while fertilization increased biomass production by 36%. Mixtures were on average more productive than expected from their monocultures (relative yield total, RYT>1) and showed positive net diversity effects (NE: +34% biomass increase; mixture minus mean monoculture biomass). Both trait-independent complementarity effects (TICE: +21%) and dominance effects (DE: +12%) positively contributed to net diversity effects, while trait-dependent complementarity effects were minor (TDCE: +1%). Shading did not alter diversity effects and overyielding. Fertilization decreased RYT and the proportion of biomass gain through TICE and TDCE, while DE increased. Diversity effects did not increase with species richness and were independent of functional group or growth stature composition. Trait-based analyses showed that the dominance of species with root and leaf traits related to resource conservation increased TICE. Traits indicating the tolerance of shade showed positive relationships with TDCE. Large DE were associated with the dominance of species with tall growth and low diversity in leaf nitrogen concentrations. Our field experiment shows that positive diversity effects are possible in grass-forb mixtures irrespective of differences in light availability, but that the chance for the complementary use of resources increases when nutrients are not available at excess.

## Introduction

Experimental studies have repeatedly shown that higher species or functional group richness increases primary productivity in grassland ecosystems and thus influences ecosystem processes [1–3]. Positive diversity-productivity relationships and overyielding, i.e. a higher productivity of mixtures than expected from the average productivity of the component species in monocultures [4], are commonly explained by two mechanisms, which are not mutually exclusive. The complementarity effect hypothesis is based on the assumption that different species complement each other in the acquisition of resources, thereby decreasing interspecific competition and increasing total resource use in space and time, resulting in higher mixture productivity [5]. The selection effect hypothesis explains positive diversity-productivity relationships by the greater probability of more diverse communities to contain a particularly dominant and productive species [6,7]. Both hypotheses propose distinct ecological mechanisms, which are controlled by the functional composition of the communities: complementarity effects depend on the interactions between functionally different species, while selection effects require the presence of a species with particular functional characteristics [8].

Functional groups are thought to capture the most relevant functional differences among species [9]. In grassland ecosystems, commonly distinguished functional groups are grasses, non-legume forbs and N_2_ fixing legumes [10–12]. Indeed, the combination of different functional groups has been shown to increase complementarity effects in experimental grasslands, which were mostly due to the presence of legumes (e.g. [2,13]). *A priori* defined functional groups often show large variation within groups [14,15] and it is still uncertain which functional traits are most relevant in explaining higher biomass production of mixtures. Hence, recent approaches stress the importance of continuous variables that quantify the trait composition of a community [16]. As such, both community weighted mean traits (CWM), which reflect the dominance of trait values [17] and indices of trait diversity, which quantify trait dissimilarity among species, have been proven as suitable predictors of aboveground biomass production in semi-natural and experimental grasslands [18–22]. For example, a study across 29 grassland diversity experiments using trait diversity alone as predictor for community biomass production has shown that diversity in traits related to nitrogen acquisition and use (leaf nitrogen concentrations, N_2_ fixation) and light competition (plant height) have the greatest predictive power for biomass production [21]. Using both community-weighted means of trait values and trait diversity, another study in the Jena Experiment has found that the dominance of particular trait values (specifically tall growth and high leaf nitrogen concentrations) is more important than trait diversity for high community biomass production [22].

Light and nutrient availability are among the most important factors limiting plant productivity in temperate grasslands [23]. The size-asymmetry of competition for aboveground resources is generally accepted [24,25]. Competition for soil resources is thought to be more size-symmetric [26] although it has been suggested that size-asymmetry in competition for belowground resources possibly occurs when soil resources are heterogeneously distributed [27,28]. Apart from size-related differences in competitive abilities, environmental factors, i.e. the external supply of resources, are likely to control the nature and intensity of plant interactions. For example, fertilization may increase asymmetric competition for light by increasing productivity, when tall and fast-growing species reduce light supply for smaller species growing deep in the canopy [29]. Thus, positive selection effects are likely to increase under fertilization, but it also has been discussed that positive complementarity effects may occur when increased soil fertility promotes light partitioning by accentuating species differences in height and growth forms [30]. However, it is also possible that a greater diversity in root characteristics increases the chance for a complementary use of soil resources in unfertile conditions when competition for belowground resources prevails [31].

Fertilization has been shown to increase diversity effects and overyielding in several experiments [30,32,33]. However, it also has been reported that the impact of soil fertility on diversity effects varies dependent on the involved species and their abundances [31,34,35], the amount of added fertilizer [34,36] and the manipulation of other resources such as light [30] or CO_2_ [33]. So far to our knowledge, only a single biodiversity experiment has crossed the manipulation of nutrient availability by fertilization with light availability by shading. This experiment has shown that overyielding is largest under increased soil fertility in full light, but the experimental species pool was restricted to annual forb species [30].

Here, we present results of a field experiment based on two independent pools of four perennial grassland species, each consisting of two species from different functional groups (grasses and forbs) and representing two growth statures (small and tall). We grew these species in monocultures, two-species and four-species mixtures at different light and nutrient availability. Using data on aboveground biomass production collected in the second year of treatment application, we quantified overyielding and calculated diversity effects applying the tripartite partitioning suggested by Fox [37], which differentiates between trait-independent complementarity effects (TICE; niche differentiation and/or facilitative interactions) and separates selection effects into dominance effects (DE; biomass gain and dominance of high-yielding monoculture species in mixture at the expense of other species) and trait-dependent complementarity effects (TDCE; biomass gain of high-yielding monoculture species in mixture without affecting other species). We asked the following questions:

1. How do light and nutrient availability and their interaction alter the relative importance of complementarity and selection effects in explaining diversity effects in grass-forb mixtures, and what are the consequences for diversity effects and overyielding at different resource supply? We predicted that trait-independent and trait-dependent complementarity effects decrease and dominance effects increase with fertilization due to a shift from prevailing competition for soil resources to greater importance of light competition. We also predicted that the effects of fertilization on diversity effects would be lower under shading, when reduced light availability generally decreases productivity and nutrients are available at excess. Consequently, we hypothesized that diversity effects are greater without shading, while the expected shift from complementarity to stronger dominance effects implies that the functional composition of the plant communities is important for varying diversity effects dependent on fertilization.
2. Do the effects of light and nutrient availability on diversity effects depend on functional group or growth stature composition? We predicted that positive dominance effects are greater in mixtures of tall and small species due to asymmetric light competition and that these effects are magnified under fertilization and less important under shading. Furthermore, we hypothesized that positive trait-independent complementarity effects are higher in grass-forb mixtures compared to mixtures with species of a single functional group irrespective of light and nutrient availability.
3. Are there general relationships between diversity effects and functional trait composition (community mean traits, trait diversity) and which traits are associated with increased complementarity and selection effects? We predicted that trait-independent complementarity effects are positively related to greater diversity in traits related to the acquisition of above- and belowground resources. We hypothesized that dominance effects occur if the dominance of particular trait values accentuates the asymmetry in the acquisition of resources, while trait-dependent complementarity effects rely on the dominance of trait values, which do not increase asymmetry in resource competition while being related to efficient resource acquisition.

## Material and Methods

### Experimental design

The field experiment was established in 2011. No specific permission was required for the described field studies. The field site used for the experiment is a former agricultural site located at the Field Station of the Helmholtz Centre for Environmental Research (UFZ) in Bad Lauchstädt (Saxony-Anhalt, Germany; 51°23'38'' N, 11°52'45'' E, 118 m a.s.l.). The site is not protected in any way, and our study does not involve endangered or protected species in Germany. The soil of the experimental site is a chernozem developed from loess [38]; soil texture is loamy sand (0–30 cm depth, for additional soil properties see S1 Table). The region is characterized by a mean of 492 mm for annual precipitation and 9.5°C for air temperature (1981–2010; weather data from intensive monitoring experiment in Bad Lauchstädt, working group C/N dynamics, UFZ, http://www.ufz.de/index.php?de=940).

Four grass and four forb species with a perennial life cycle and native to semi-natural temperate grasslands (Arrhenatherion community, [39]) were chosen for the experiment and randomly assigned to two species pools (Table 1). Each experimental species pool included an inherently small-statured and a tall-statured species of each functional group. The replication of the experiment with two independent species pools aimed to exclude large species identity effects because we wanted to focus on functional composition with respect to functional group and growth stature differences.

**Table 1:** Species pools of the biodiversity experiment. Listed are studied species and taxonomy, growth height [64], assignment to functional groups (grasses or forbs), different growth statures (small or tall), and species pools.

Species	Family	Height (cm)	Stature	Functional group	Species pool
*Anthoxanthum odoratum* L.	Poaceae	20–50	small	grass	A
*Lolium perenne* L.	Poaceae	10–60	small	grass	B
*Arrhenatherum elatius* (L.) J. & C.Presl	Poaceae	60–120	tall	grass	A
*Dactylis glomerata* L.	Poaceae	50–150	tall	grass	B
*Plantago lanceolata* L.	Plantaginaceae	10–50	small	forb	A
*Prunella vulgaris* L.	Lamiaceae	5–30	small	forb	B
*Centaurea jacea ssp*. *jacea* L.	Asteraceae	15–80	tall	forb	A
*Knautia arvensis* (L.) Coulter	Dipsacaceae	30–80	tall	forb	B

The experiment consisted of 96 plots of 2 × 2 m size, encompassing monocultures of each species and all possible two-species combinations that were replicated four times, while the four-species combinations of each species pool were established with eight replicates. Plots were arranged in eight blocks, each containing an equal number of plots per species-richness level (four monocultures, six two-species mixtures and two four-species mixtures) and per species pool ensuring that individual species occurred an equal amount of times (= three times) in each block. Seeds from the closest possible regional provenance were purchased from a commercial supplier (Rieger-Hoffman GmbH, Blaufelden-Raboldshausen, Germany). Plots were sown with a total initial density of 1000 viable seeds per m^2^ (adjusted for germination rates determined in laboratory tests) on 5 April 2011. In the mixtures, individual species were sown at equal proportions. After a first mowing in September 2011, all plots were re-sown on 4 October 2011 with 500 viable seeds per m^2^ to imitate a more diverse age structure of plant populations.

In the second experimental year, one replicate per species composition (or two replicates in case of the four-species mixtures) was assigned to the following “environments” manipulating nutrient and light availability: (F-S-) no fertilization, no shading, (F-S+) no fertilization, shading, (F+S-) fertilization, no shading, and (F+S+) fertilization, shading. In four blocks, wooden scaffoldings were installed reaching from ground level to 2.10 m height. In mid-April, one layer of green shading cloth (polyethylene, aperture size 2 × 10 mm, Hermann Meyer KG, Rellingen, Germany) was attached to these scaffoldings and fastened to the ground on all sides until removal in mid-September of each experimental year to simulate shading. Photosynthetically active radiation (PAR) in shaded blocks was reduced by 55% during daytime compared to blocks without shading (based on continuous half-hourly measurements with SPK125, PAR Quantum Sensor; Skye Instruments Ltd, UK). The arrangement of blocks in the field ensured that shade scaffoldings did not interfere with light availability in surrounding blocks. In each block, an equal number of plots per species-richness level were selected for the nutrient addition treatment. Fertilizer was administered in granular form (commercially available slow release NPK fertilizer 120:52:100 kg ha^-1^ yr^-1^) and was applied in two equal portions in spring (mid-March) and after first mowing (mid-June). The amount of added nutrients is equivalent to usual fertilizer intensities in agriculturally managed semi-natural grasslands in Europe [40].

All plots were mown in early June and mid-September and mown plant material was removed to imitate the regionally common management of extensive hay meadows. Species not being part of the original plot species combinations were weeded out by hand in regular intervals when the vegetation was low and the canopy not completely closed (early in the growing season, after first mowing and after second mowing).

### Data collection

In the second year of treatment applications, aboveground biomass was harvested at estimated peak biomass prior to the mowing in spring (27–29 May 2013) and summer (26–29 August 2013). Plant material was cut 3 cm above ground level in two randomly allocated rectangles (0.2 × 0.5 m) excluding the outer 0.4 m to the plot margin. All samples were sorted to sown species; detached dead plant material and weeds were separated from the samples. After drying for 48 h at 70°C, samples were weighed and community- and species-level annual aboveground biomass production (g m^-2^) was attained by summing up both harvests.

### Data analyses

#### Calculations of measures of overyielding and diversity effects

Overyielding in relative terms [41] was quantified as relative yield total (RYT). The relative yield (RY_i_) of a species i is the quotient of a species' mixture biomass (Y_iO_) and its monoculture biomass (BM_i_). The RYT of a mixture is the sum of the relative yields of each component species:

$$RYT=\sum_{i=1}^{S}RY_{i}$$ (1)

A RYT > 1 indicates that the proportional increase in the biomass of particular species is greater than the possible proportional decrease in the biomass of other species, i.e. a mixture outperforms the average of its component monocultures. It is directly associated to non-transgressive overyielding

$$D_{mean}=\left(Y_{O}-\bar{BM}\right)/\bar{BM}$$ (2)

where Y_o_ is the observed biomass of a given mixture and $\bar{BM}$ is the average monoculture biomass of all species in this mixture (*D*_*mean*_ = RYT-1; [4]). Transgressive overyielding, i.e. a higher productivity of a mixtures than its most productive monoculture, was calculated by comparing the biomass of a given mixtures to the maximum monoculture biomass of the species in that mixture [4]

$$D_{max}=\left(Y_{O}-\text{max}\left(BM_{i}\right)\right)/\text{max}\left(BM_{i}\right)$$ (3)

The relative yields (RY_i_) of individual species corrected for sowing proportions (i.e. multiplied by species number S) were used to compare the performance of species and their contribution to overyielding. Species perform better in mixture than in monoculture if (RY_i_ *S) > 1, their performance in mixture does not differ from monoculture if (RY_i_ * S) = 1, and values of (RY_i_ * S) < 1 indicate that species perform worse in mixture than in monoculture.

The tripartite partitioning method [37], which is an extension of the additive partitioning method by Loreau and Hector [42] was applied to assess diversity effects on aboveground biomass production in absolute terms. The net diversity effects (NE) is the difference between observed (*Y*_*O*_) and expected (*Y*_*E*_) biomass in mixtures and also the sum of three additive components, the trait-independent complementarity effect (TICE), the trait-dependent complementarity effect (TDCE) and the dominance effect (DE). The trait-independent complementarity effect (TICE), which equals the complementarity effect (CE) according to Loreau and Hector [42], is positive (negative) if species biomass in a mixture is on average higher (lower) than the average of species biomass in monocultures and is calculated as

$$TICE=\;S\;\Delta \bar{RY}\;\bar{BM_{\;}}$$ (4)

where $\Delta \bar{RY}$ is the average Δ*RY*_i_ (= difference between the observed relative yield and the expected relative yield of species i) and $\bar{BM}$ is the average monoculture biomass of all species growing in this mixture. The selection effect (SE) according to Loreau and Hector [42] is partitioned into two covariance terms. The dominance effect (DE)

$$DE=\;S\;cov\;\left(BM_{i},\frac{\Delta RY_{i}}{RYT}\right)$$ (5)

is the covariance between species`biomass in monoculture (*BM*_*i*_) and the differences between species`observed and expected relative yields divided by relative yield totals (*ΔRY*_*i*_*/RYT*). The DE is positive if species with high monoculture biomass dominate a mixture at the expense of species with low monoculture biomass. A negative DE indicates that a mixture is dominated by a species with low monoculture biomass at the expense of others [37]. The trait-dependent complementarity effect (TDCE)

$$TDCE=\;S\;cov\;\left(BM_{i},RY_{o}-\frac{RY_{o}}{RYT}\right)$$ (6)

is the covariance between species`biomass in monoculture (*BM*_*i*_) and the difference between species observed relative yields (*RY*_*o*_) and it observed contribution to RYT (*RY*_*o*_*/RYT*). The TDCE is positive (negative) if species with high (low) monoculture biomass have high relative yields, but not at the expense of other species.

In all calculations, the observed species biomass in the monoculture of a particular “environment”, i.e. resource treatment combination, was used to derive the expected biomass in the mixtures of the same species × resource treatment combination.

#### Functional trait composition of the mixtures

To evaluate if trait-independent complementarity (TICE), trait-dependent complementarity (TDCE) and dominance effects (DE) derived from tripartite partitioning could be explained by the functional trait composition of the mixtures, we selected pairs of above- and belowground traits, which are known to be related to resource uptake above- and belowground (specific leaf area = SLA, specific root length = SRL), to plant stature (maximum stretched shoot length = H_max_, weighted mean depth of standing root biomass = WMD) and nitrogen acquisition and use (leaf nitrogen concentration = LNC, root nitrogen concentration = RNC). To account for environment-induced trait variation, data obtained in each of the studied “environments”, i.e. resource treatment combinations, were used for the characterisation of functional trait composition. Species-specific traits for root characteristics were taken from monocultures [43], while values for aboveground traits were averaged for each species across all communities per “environment”, see Supporting Information (S1 File) for details on trait measurements.

Rao's quadratic entropy (Rao's Q; [44]), which quantifies trait diversity (FD) as the sum of pairwise distances between species weighted by their relative abundance, was assessed separately for each of the chosen six traits using the R package *FD* [45]

$$FD=\overset{S}{\underset{i=1}{\sum }}\overset{S}{\underset{j=1}{\sum }}p_{i}p_{j}d_{ij}$$ (7)

where *S* is the number of species in the community, *p*_*i*_ and *p*_*j*_ are the relative abundances of species *i* and *j*, and *d*_*ij*_ is the trait distance between species *i* and *j* in the community.

Community weighted means of trait values were computed as

$$CWM=\sum_{i=1}^{S}{{}_{p_{i}t_{i}}}_{}{}_{}$$ (8)

where *S* is the number of species in the community, *p*_*i*_ is the relative abundance of the *i*-th species in the community and *t*_*i*_ is the trait value of species *i* [17]. Species proportions in aboveground biomass were used as relative abundances for the calculation of both measures.

#### Statistical analyses

All statistical analyses were performed with the statistical software R 3.2.3 [46]. Linear mixed-effects models (R package *lme4*; [47]) were applied to analyse the effects of the experimental factors on the measured variables. For community-level analyses the random structure of the models included independent terms for block and mixture identity (= identity of each particular species combination). Starting from a constant null model, the fixed effects were added in the following order: shade (two factor levels: no shade, shade), fertilization (two factor levels: no fertilization, fertilization), species richness (SR; log-linear term), functional group composition (FG; three factor levels: pure grass communities, pure forb communities, grass-forb communities), growth stature composition (GS; three factor levels: pure small-species communities, pure tall-species communities, small/tall-species communities) followed by all possible two-way and three-way interactions. In order to evaluate the statistical significance of the fixed effects, we used the approximate F statistics (Kenward-Roger approximation) with type I SS as provided in the R package *lmerTest* [48]. A likewise approach was applied to species-level data (species biomass, relative yields). Species biomasses (BM_i_) and relative yields (RY_i_) were multiplied by the number of sown species in the mixtures prior to statistical analyses to account for decreasing sown proportions of individual species at increasing species richness. The structure of random terms for species-level analyses consisted of block, plot nested in block, mixture identity and species identity. Additionally, terms for the functional composition of the mixtures were replaced by terms for functional group identity (FG-ID) and growth stature identity (GS-ID) and the respective interactions. The *difflsmeans* function (R package *lmerTest*; [48]) was applied to identify significant differences between communities of different functional composition (functional group and growth stature composition) in community-level analyses and among species in species-level analyses in post-hoc tests of the respective models analysing the differences between least squares.

The lack of identical replicates of each species combination in the different resource treatments, which would allow testing for mixture identity effects, is one shortcoming of our experimental design (and many other biodiversity experiments). In addition, our experiment had only a small number of four-species mixtures compared to the two-species mixtures, which could lower the robustness of analyses with respect to species richness effect.

In order to assess which functional trait characteristics best accounted for variation in TICE, TDCE and DE, multimodel inference (R package *MuMIn*; [49]) was applied. Correlation coefficients among candidate predictor variables (CWM and FD based on different traits as described above) were generally low and did not exceed r = 0.7. Therefore, it was assumed that collinearity is not a major problem for modelling [50] and all measures of trait composition were entered as predictor variables into a global model with block as random term. The most appropriate combination of predictor variables was automatically selected by comparing Akaike information criteria (AIC) of models with all possible combinations of fixed effects, where the number of fixed effects was restricted to a maximum of three to avoid overfitting. To account for model selection uncertainty, 95%-confidence intervals for coefficients associated with predictors included in models with very similar AIC were estimated by averaging over models with delta < 4. The contribution of each potential model to the averaged parameter estimates is proportional to the Akaike model weight. If a predictor is not incorporated in the model, the corresponding parameter estimate is set to zero. The relative importance of predictors was calculated as the sum of Akaike model weights of models where the respective predictors appear [51]. Trait diversity (for all traits) and community-weighted mean traits (for SRL, RNC, LNC) were transformed to their natural logarithms to fulfil the assumptions of linear mixed-effect models. Two plots with a two-species mixture were excluded from these analyses because one species was missing and therefore FD could not be calculated.

## Results

### Community biomass production

**Fig 1:**
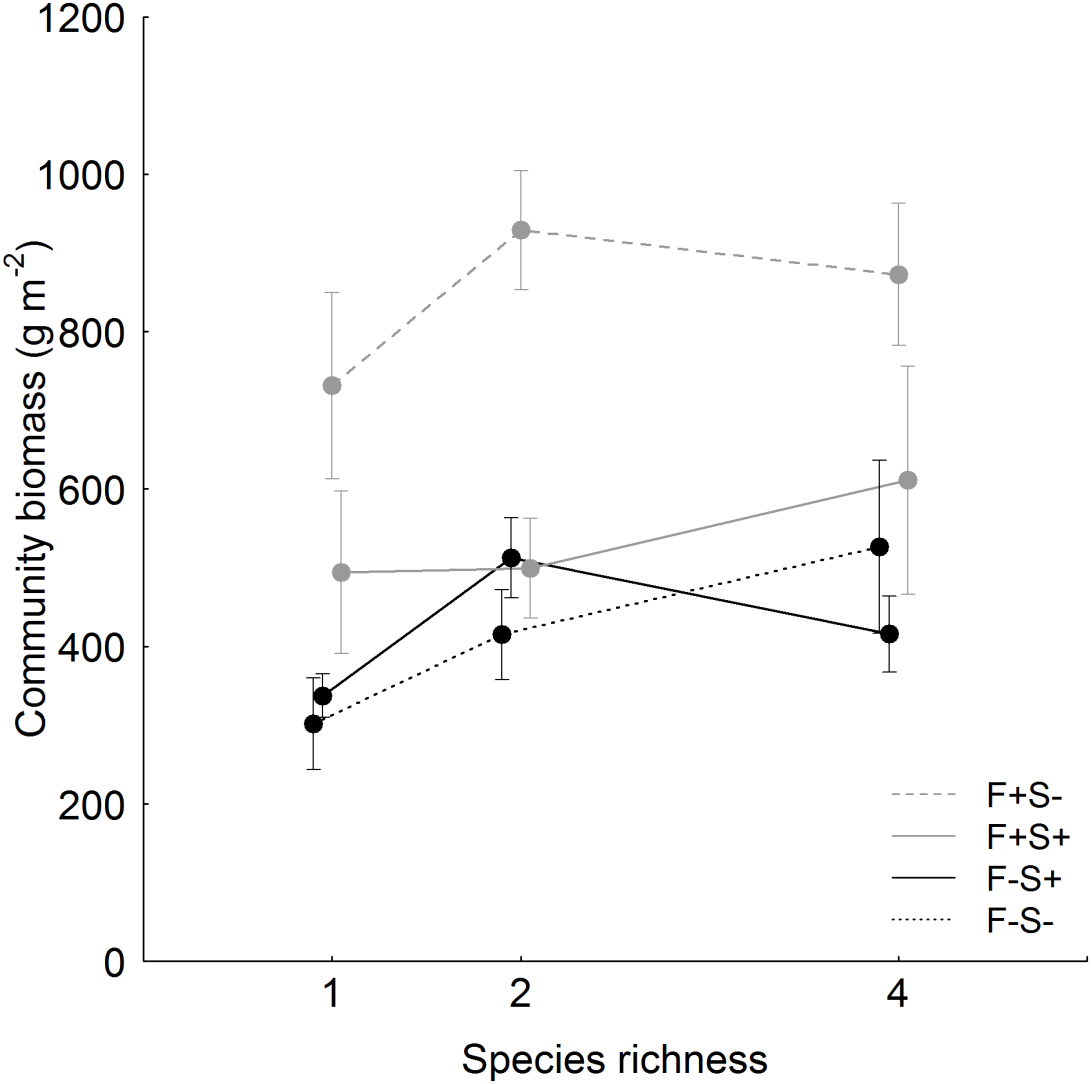
Effects of resource availability on aboveground biomass production in communities of different species richness. Values are means (± 1 SE) per resource treatment and species-richness level. Values are staggered along the x-axis for enhanced clarity. Treatments manipulating resource availability are abbreviated with: F-S- = no fertilization, no shading, F-S+ = no fertilization, shading, F+S- = fertilization, no shading, and F+S+ = fertilization, shading.

Shading had negative effects on aboveground biomass production, while fertilization increased aboveground community biomass (Table 2). Positive effects of fertilization on community biomass production were abated under shading (Fig 1). Unshaded fertilized communities produced significantly more biomass (mean ± SD: 915 ± 239 g m^-2^) than shaded fertilized communities (511 ± 233 g m^-2^), and unfertilized communities with shading (490 ± 169 g m^-2^) and without shading (443 ± 202 g m^-2^).

**Table 2:** Summary of linear mixed-effects model analyses for community biomass production, relative yield totals (RYT), transgressive overyielding (D_max_), net diversity effects (NE), trait-independent complementarity effects (TICE), trait-dependent complementarity effects (TDCE) and dominance effects (DE). Plant communities of different species richness, functional group and growth stature composition were grown at different levels of resource availability manipulating light supply by shading and nutrient supply by fertilization.

Source of variation		Community biomass				RYT				D_max_							
	df	MS	F	p		MS	F	p		MS	F	p					
Shade	1	**0.896**	**8.32**	**0.048**	**↓**	0.191	1.30	0.326		0.002	0.01	0.925					
Fertilizer	1	**5.324**	**49.44**	**<0.001**	**↑**	**1.003**	**6.79**	**0.014**	**↓**	**1.314**	**7.82**	**0.009**	**↓**				
Species richness (SR)	1	**0.535**	**4.97**	**0.043**	**↑**	0.004	0.03	0.872		0.225	1.34	0.320					
Functional group composition (FG)	2	0.059	0.55	0.589		0.012	0.08	0.924		0.081	0.48	0.631					
Growth stature composition (GS)	2	**0.654**	**6.07**	**0.011**		0.071	0.48	0.633		0.005	0.03	0.972					
Shade x Fertilizer	1	**3.337**	**30.99**	**<0.001**		0.175	1.19	0.285		**0.750**	**4.46**	**0.044**					
Shade x SR	1	0.105	0.97	0.328		0.010	0.07	0.796		<0.001	0.00	0.965					
Fertilizer x SR	1	0.116	1.08	0.304		0.024	0.16	0.689		0.015	0.09	0.765					
Shade x FG	2	0.122	1.13	0.331		0.328	2.22	0.127		0.177	1.05	0.363					
Fertilizer x FG	2	0.165	1.53	0.228		0.016	0.11	0.899		0.090	0.53	0.592					
Shade x GS	2	0.120	1.11	0.337		0.071	0.48	0.622		0.159	0.95	0.400					
Fertilizer x GS	2	0.033	0.31	0.738		0.097	0.66	0.526		0.174	1.04	0.367					
Shade x Fertilizer x SR	1	0.060	0.56	0.460		0.230	1.56	0.222		0.312	1.86	0.184					
Shade x Fertilizer x FG	2	0.026	0.24	0.789		0.016	0.11	0.899		0.001	0.01	0.993					
Shade x Fertilizer x GS	2	**0.479**	**4.45**	**0.016**		0.215	1.45	0.250		0.206	1.22	0.308					
Source of variation			NE			TICE				TDCE				DE			
	df	MS	F	p		MS	F	p		MS	F	p		MS	F	p	
Shade	1	78957	1.87	0.255		56326	1.43	0.305		389	0.38	0.576		4221	0.48	0.540	
Fertilizer	1	22749	0.54	0.469		88247	2.24	0.146		**6189**	**6.08**	**0.020**	**↓**	**50587**	**5.76**	**0.024**	**↑**
Species richness (SR)	1	2821	0.07	0.809		6141	0.16	0.716		1486	1.46	0.280		1477	0.17	0.696	
Functional group composition (FG)	2	19306	0.46	0.646		21878	0.56	0.590		292	0.29	0.758		549	0.06	0.940	
Growth stature composition (GS)	2	7997	0.19	0.831		20331	0.52	0.611		1638	1.61	0.254		8018	0.91	0.438	
Shade x Fertilizer	1	102159	2.42	0.131		44828	1.14	0.295		323	0.32	0.577		8083	0.92	0.346	
Shade x SR	1	1216	0.03	0.867		168	0.00	0.948		3770	3.71	0.065		184	0.02	0.886	
Fertilizer x SR	1	1252	0.03	0.865		4533	0.12	0.737		14	0.01	0.909		798	0.09	0.765	
Shade x FG	2	65815	1.56	0.228		82550	2.09	0.142		412	0.41	0.671		1185	0.14	0.874	
Fertilizer x FG	2	41917	0.99	0.385		20417	0.52	0.602		96	0.09	0.910		3735	0.43	0.658	
Shade x GS	2	23372	0.55	0.581		9030	0.23	0.797		217	0.21	0.809		8146	0.93	0.408	
Fertilizer x GS	2	47876	1.13	0.336		21104	0.54	0.591		883	0.87	0.430		5141	0.59	0.563	
Shade x Fertilizer x SR	1	105936	2.51	0.125		50006	1.27	0.270		1184	1.16	0.290		4549	0.52	0.478	
Shade x Fertilizer x FG	2	8700	0.21	0.815		4564	0.12	0.891		639	0.63	0.542		2590	0.30	0.747	
Shade x Fertilizer x GS	2	107621	2.55	0.095		67865	1.72	0.196		327	0.32	0.727		8641	0.98	0.386	

Models were fitted by stepwise inclusion of fixed effects. Approximate F statistics (type I SS using Kenward-Roger degrees of freedom approximation) was used to assess the statistical significance of the fixed effects (p values). Significant effects are marked in bold. Arrows indicate an increase (↑) or decrease (↓) of the studied variable with shading, fertilizer addition or species richness.

Increasing species richness slightly increased aboveground community biomass. While aboveground biomass production did not change dependent on functional group composition, communities comprising only small-statured species produced less biomass than mixtures of only tall-statured species, while mixtures of both growth statures showed intermediate productivity. The combination of fertilization and shading affected community biomass production differentially, depending on growth stature composition. Communities comprising tall species, especially mixtures with only tall species, were more productive under fertilization and were less negatively influenced by shading than communities with only small species and mixtures of tall and small species.

### Species relative yields

**Fig 2:**
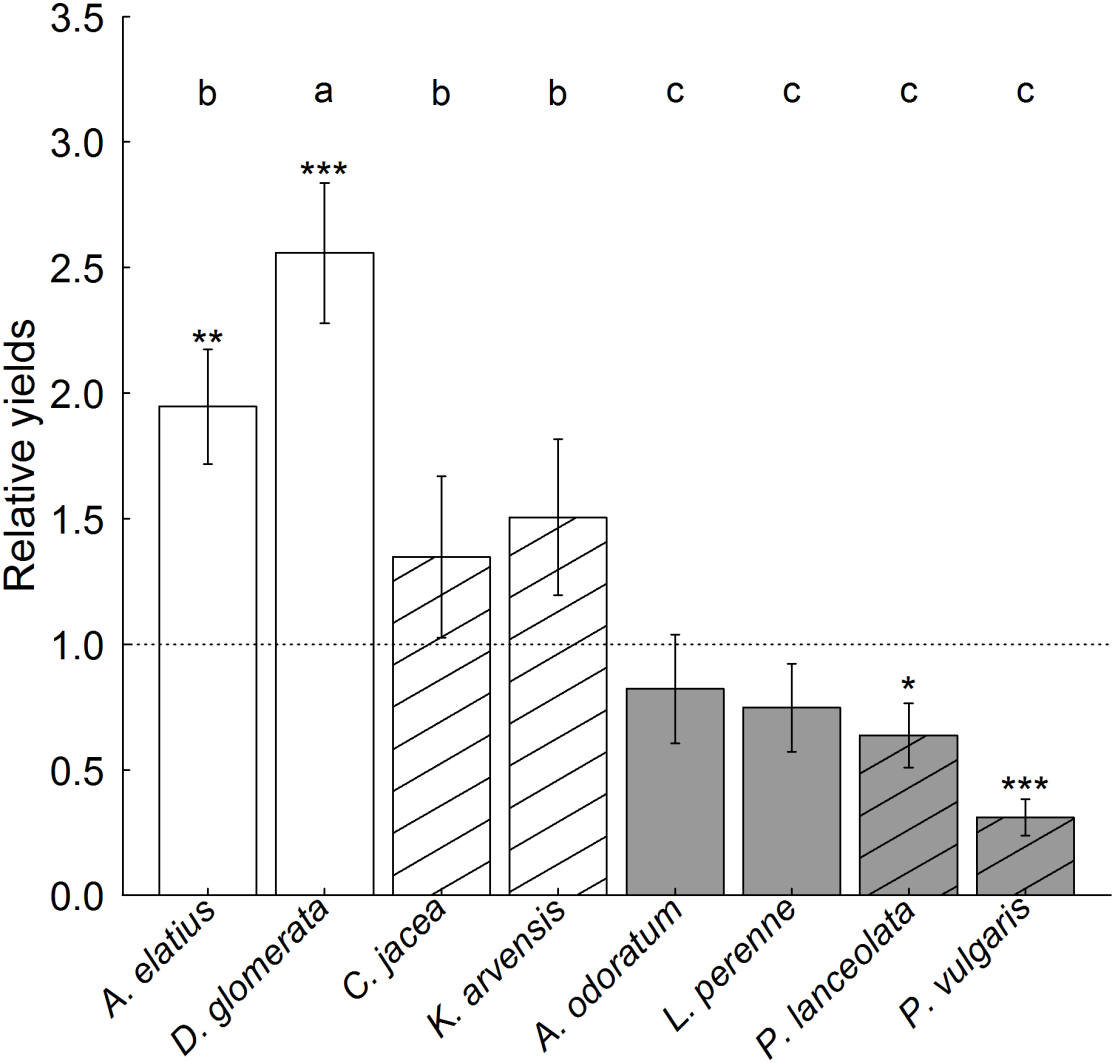
Species—level relative yields (RYs). Shown are means (± 1 SE) across communities of different species richness and grown at varying resource availability. The threshold for greater biomass production of a species in the mixtures than expected from its monoculture (RY > 1) is indicated with a dotted line. Results of overall tests for RY ≠ 1 for each species are indicated with * p ≤ 0.05, ** p ≤ 0.01 and *** p ≤ 0.001. Different letters indicate significant differences among species in their RYs. Hatched bars = forbs, open bars = grasses; filled bars = small-statured species, unfilled bars = tall-statured species.

Species relative yields (RYs) were higher without fertilization than with fertilization, yet not dependent on light availability or different between two-species and four-species mixtures (S2 Table). Grasses had higher RYs than forbs. The RYs of small-statured species were smaller than those of tall-statured species. Tall grasses (*A*. *elatius*, *D*. *glomerata*) had significantly higher biomass in mixtures than expected from their monocultures (RY > 1), while the RYs of tall forbs and small grasses did not differ from expected values and those of small forbs were even smaller than expected (Fig 2).

The effects of light availability on RYs differed between functional groups and growth statures (S2 Table). Species relative yields of forbs were lower in shaded compared to unshaded communities, whereas grass species attained higher relative yields under shading compared to full light conditions (S2 Table, S1a Fig). Differences in the RYs of tall- and small-statured species were smaller in shaded communities (S2 Table, S1b Fig). Differences in species-level biomass production were similar to those observed for species relative yields indicating that highly productive species also obtained large relative yields (S2 Table, S2 Fig).

### Non-transgressive and transgressive overyielding

**Fig 3:**
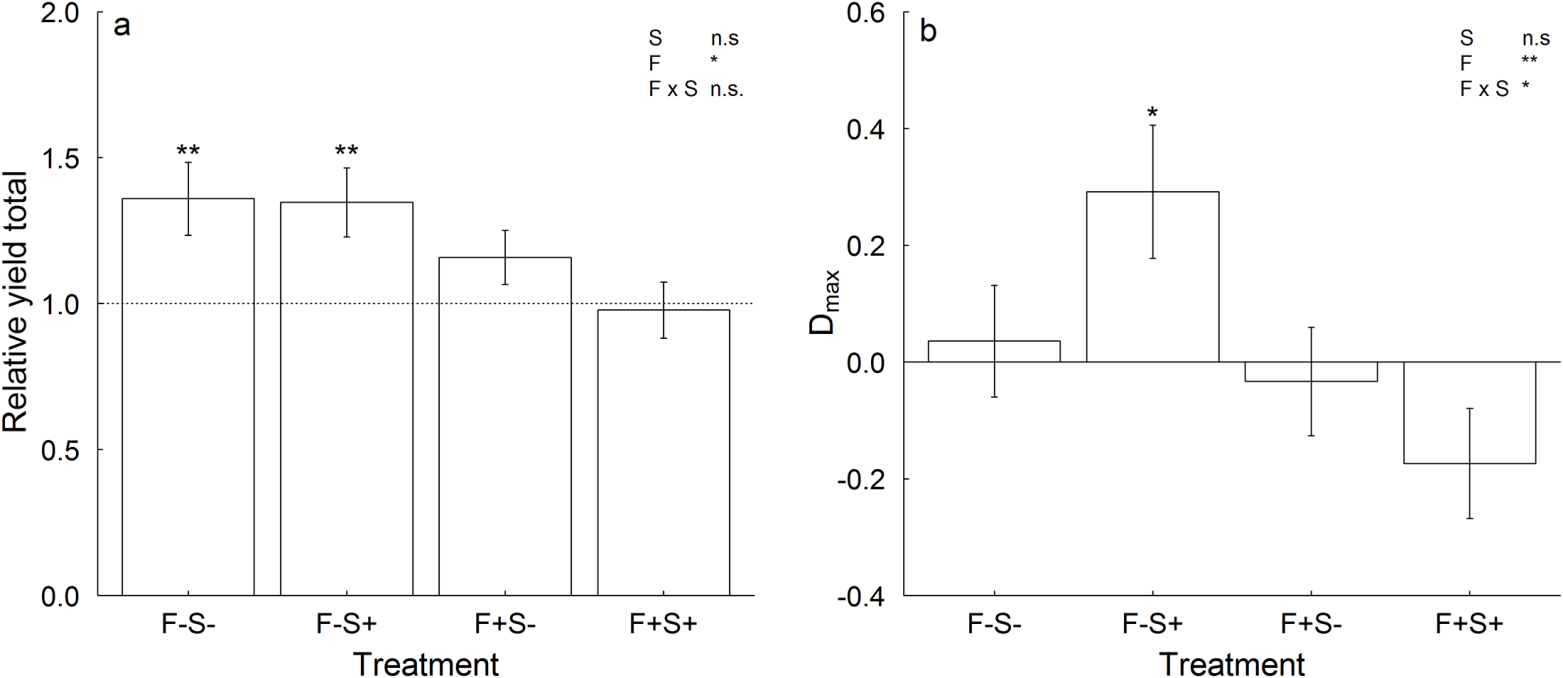
Effects of resource availability on (a) relative yield totals (RYT), and (b) D_max_. Shown are means (± 1 SE) across two- and four-species mixtures per resource treatment. Treatments manipulating resource availability are abbreviated with: F-S- = no fertilization, no shading, F-S+ = no fertilization, shading, F+S- = fertilization, no shading, and F+S+ = fertilization, shading. Results of tests for overall means of RYT ≠ 1 and D_max_ ≠ 0, respectively, for each resource treatment are indicated for different levels of significance with * p ≤ 0.05, ** p ≤ 0.01 and *** p ≤ 0.001. Levels of significance from linear mixed effects models (Table 2) for effects of shade, fertilization and their interaction are given in the upper right corner.

In total, 67% of the mixtures (N = 64) showed non-transgressive overyielding (i.e. RYT > 1). Fertilization had negative effects on relative yield totals (Table 2, Fig 3a). On average, the RYTs were > 1 without fertilization while the RYTs were not significantly different from 1 across fertilized communities. Irrespective of light and nutrient availability, RYTs did not differ depending on species richness, functional group or growth stature composition (Table 2).

Only 44% of mixtures displayed transgressive overyielding (D_max_ > 0). Transgressive overyielding also varied with resource availability (Table 2). On average, mixture productivity exceeded the most productive monoculture in the shade without fertilization (D_max_ > 0), while this was not the case in the other resource treatment combinations. D_max_ did neither depend on species richness, nor functional group and growth stature composition.

### Net diversity effects, trait-independent and trait-dependent complementarity effects and dominance effects

**Fig 4:**
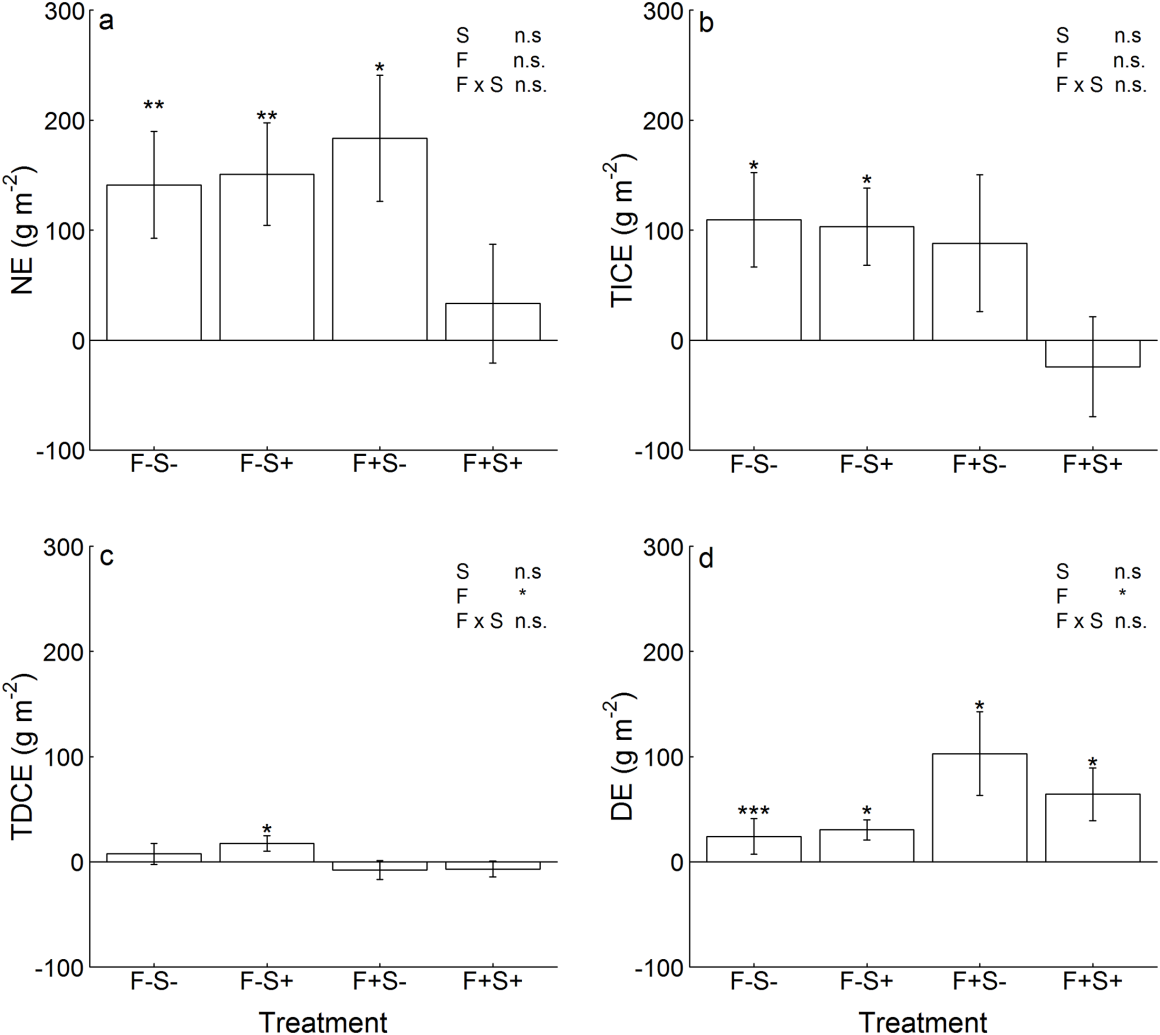
Effects of resource availability on (a) net diversity effects (NE), (b) trait-independent complementarity effects (TICE), (c) trait-dependent complementarity effects (TDCE) and (d) dominance effects (DE). Shown are means (± 1 SE) across two- and four-species mixtures per resource treatment. Treatments manipulating resource availability are abbreviated with: F-S- = no fertilization, no shading, F-S+ = no fertilization, shading, F+S- = fertilization, no shading, and F+S+ = fertilization, shading. Results of tests for overall means of diversity effects ≠ 0 for each resource treatment are indicated for different levels of significance with * p ≤ 0.05, ** p ≤ 0.01 and *** p ≤ 0.001. Levels of significance from linear mixed effects models (Table 2) for effects of shade, fertilization and their interaction are given in the upper right corner.

Light and nutrient availability, sown species richness, functional group or growth stature composition did not significantly affect NE (Table 2). The overall mean of net diversity effects (NE) was positive across all mixtures (test for overall mean > 0; p < 0.001; Fig 4a) and amounted to +128 (± 213) g m^-2^. Due to the different productivity-levels of the communities dependent on resource availability, the biomass gain in mixtures compared to the monocultures reached ~49% without fertilization irrespective of shading. Biomass gain was ~28% in fertilized unshaded mixtures and amounted to only ~13% in fertilized shaded mixtures. On average, positive net diversity effects were attributable to similar levels of positive trait-independent complementarity effects (TICE: +69 ± 193 g m^-2^) and positive dominance effects (DE: +55 ± 104 g m^-2^), while levels of positive trait-dependent complementarity effects were minor (TDCE: +3 ± 35 g m^-2^) (Fig 4b–4d). Trait-independent complementarity effects (TICE) did not vary dependent on light and nutrient availability, but varied greatly among mixtures within each resource treatment (Fig 4b). Again, the proportion of biomass gain attributable to positive TICE differed dependent on resource availability: in unfertilized mixtures it was ~35% in shaded and unshaded conditions, while it was ~16% in fertilized unshaded mixtures. On average, TICE became even negative in fertilized shaded communities (~3% biomass loss). Trait-dependent complementarity effects (TDCE) were slightly larger in unfertilized than in fertilized mixtures, irrespective of shading (Table 2, Fig 4c). Positive TDCE were significant in non-fertilized shaded mixtures and led to a biomass gain of ~6%, while TDCE did not significantly contribute to diversity effects in non-fertilized shaded mixtures (~3% biomass gain) and in fertilized mixtures (~1% biomass loss irrespective of shading). In contrast, dominance effects (DE) were slightly larger in fertilized than in non-fertilized mixtures, irrespective of shading (Table 2, Fig 4d). The biomass gain due to positive DE amounted to ~10% in non-fertilized mixtures while it was ~13% in fertilized non-shaded mixtures and ~16% in fertilized shaded mixtures. Sown species richness, functional group or growth statures composition had no additional effects on TICE, TDCE and DE (Table 2).

### Relationships between functional trait composition and trait-independent and trait-dependent complementarity effects and dominance effects

Trait-independent complementarity effects (TICE) decreased with increasing diversity in leaf nitrogen concentrations (negative correlation with FD_LNC_, S3 Table). Combined analyses of all predictor variables showed that CWM_RNC_, CWM_SRL_ and FD_LNC_ (all negative effects) in combination best explained variation in TICE, whereby the relative importance of FD_LNC_ was greater than that of CWM_RNC_ and CWM_SRL_ (Table 3, S4 Table). Trait-dependent complementarity effects (TDCE) increased with decreasing community-weighted means in leaf nitrogen concentrations (negative correlation with CWM_LNC_) and increasing community-weighted means in root depth distribution (positive correlation with CWM_WMD_; S3 Table). In analyses with all predictor variables, CWM_SLA_ (positive effects) and CWM_LNC_ and CWM_SRL_ (both negative effects) together best explained variation in TDCE (Table 3, S4 Table). Dominance effects were positively related with community-weighted means in shoot height (CWM_Hmax_) and correlated negatively with diversity in specific root length (FD_SRL_), leaf and root nitrogen concentrations (FD_LNC_, FD_RNC_) and root depth distribution (FD_WMD_; S3 Table). In the combined analyses with all predictor variables, FD_LNC_ (negative effects) was the best predictor variable for DE, while the relative importance of CWM_Hmax_ (positive effects) was of lower explanatory power (Table 3, S4 Table).

**Table 3:** Results of linear mixed-effects model analyses predicting trait-independent complementarity effects (TICE), trait-dependent complementarity effects (TDCE) or dominance effects (DE) from trait-based predictors.

Trait-based predictors	Estimate	SE	Lower 95% CI	Upper 95% CI	Relative variable importance
**Trait-independent complementarity effects (TICE)**					
Community-weighted mean traits					
CWM_SLA_	0.06	1.70	-3.34	3.46	0.06
CWM_SRL_	-41.26	59.49	-158.75	76.22	**0.43**
CWM_LNC_	-2.34	31.27	-64.86	60.19	0.06
CWM_RNC_	-161.60	134.63	-427.81	104.62	**0.71**
CWM_Hmax_	-0.04	0.34	-0.72	0.65	0.07
CWM_WMD_	0.45	6.49	-12.43	13.33	0.09
Trait diversity					
FD_SLA_	-1.02	7.07	-15.10	13.07	0.08
FD_SRL_	-0.61	6.43	-13.46	12.24	0.07
FD_LNC_	-36.56	15.54	-67.54	-5.58	**0.98**
FD_RNC_	0.01	3.20	-6.40	6.43	0.06
FD_Hmax_	-1.49	7.98	-17.28	14.31	0.09
FD_WMD_	1.32	6.37	-11.33	13.97	0.09
**Trait-dependent complementarity effects (TDCE)**					
Community-weighted mean traits					
CWM_SLA_	4.901	2.77	-0.58	10.39	**0.89**
CWM_SRL_	-12.791	14.03	-40.49	14.91	**0.55**
CWM_LNC_	-89.435	47.64	-183.86	4.99	**0.89**
CWM_RNC_	-	-	-	-	-
CWM_Hmax_	-	-	-	-	-
CWM_WMD_	1.538	2.89	-4.17	7.25	0.30
Trait diversity					
FD_SLA_	-	-	-	-	-
FD_SRL_	-	-	-	-	-
FD_LNC_	-	-	-	-	-
FD_RNC_	-	-	-	-	-
FD_Hmax_	-	-	-	-	-
FD_WMD_	-	-	-	-	-
**Dominance effects (DE)**					
Community-weighted mean traits					
CWM_SLA_	-0.07	1.31	-2.69	2.55	0.11
CWM_SRL_	-1.48	10.64	-22.72	19.75	0.12
CWM_LNC_	-7.25	30.46	-67.75	53.25	0.14
CWM_RNC_	6.30	26.04	-45.44	58.04	0.14
CWM_Hmax_	0.25	0.54	-0.81	1.31	**0.27**
CWM_WMD_	0.79	3.67	-6.50	8.08	0.13
Trait diversity					
FD_SLA_	1.28	5.55	-9.76	12.32	0.14
FD_SRL_	-1.70	6.36	-14.34	10.93	0.15
FD_LNC_	-23.66	8.26	-40.16	-7.15	**1.00**
FD_RNC_	-0.46	2.86	-6.16	5.23	0.12
FD_Hmax_	1.73	5.62	-9.40	12.86	0.17
FD_WMD_	-1.25	4.52	-10.22	7.72	0.16

Shown are standardized parameter estimates, standard error (SE), 95% confidence intervals (CI) and relative variable importance after full model averaging of models with delta <4 including up to three predictor variables (see for the best models in S4 Table in Supporting Information). Abbreviations are: CWM = community-weighted mean traits, FD = trait diversity, Hmax = shoot length, LNC = leaf nitrogen concentration, RNC = root nitrogen concentration, SLA = specific leaf area, SRL = specific root length, WMD = weighted mean depth of root biomass distribution.

## Discussion

Positive diversity-productivity relationships have been observed in many grassland diversity experiments (e.g. [2,8,13]). Legume presence has been repeatedly reported as a main factor increasing biomass production via facilitation [2,52], but increased mixture productivity has also been observed in biodiversity experiments excluding legumes [53]. This is in line with our results showing that biomass production increased with increasing species richness. Mixtures were on average more productive than expected from monocultures (RYT > 1). However, RYT and diversity effects did not increase from the two- to the four-species mixtures and their extent varied with the availability of soil resources.

### How do light and nutrient availability alter overyielding and diversity effects in grass-forb mixtures?

Although total productivity levels increased with fertilization in our experiment, the relative biomass increase in the mixtures compared to the monocultures was greater without fertilization as indicated by larger RYT (Fig 3a). These results concur with our expectations that proportional biomass gain through trait-independent complementarity effects (TICE) decreases with increasing nutrient availability due to released competition for soil resources. In line with our results, lower TICE have also been observed under fertilization in other grassland diversity experiments [31,54]. In contrast, fertilization did not affect TICE in an experiment with early- and mid-successional species [34]. It has been suggested [33] that diversity effects due to the complementary use of soil resources might only be visible if a certain level of nutrient availability is achieved because possible strategies for resource capture and use at low nutrient availability are limited. Conversely, it is also possible that a complementary use of soil resources becomes dispensable, when nutrients are available at excess. For example, reduced complementarity effects have been found at very high levels of fertilization (≥ 400 kg N ha^-1^ yr^-1^) [31,36]. In contrast, the amount of added fertilizer in our experiment was more similar to the study by Wacker et al. ([34]; 80, 160 and 240 kg N ha^-1^ yr^-1^), who did not find effects of fertilization on TICE. However, the chernozem at our experimental site represents a nutrient-rich substrate, where fertilization could result in nutrient excess.

In line with our expectations, dominance effects (DE) increased with fertilization. Other experiments have shown positive effects of fertilization on selection effects [31,34,35], but did not separate them into dominance effects and trait-dependent complementarity effects as suggested by Fox [37]. Diversity effects attributable to trait-dependent complementarity effects (TDCE) were near negligible. However, in line with our hypothesis TDCE decreased with fertilization and were only positive across all mixtures, when unfertilized communities were shaded (Fig 4c). As D_max_ was highly positively correlated with TDCE (r = 0.752, p < 0.001; N = 64) in our experiment, we also found that most mixtures in this treatment showed transgressive overyielding. One possible explanation for the frequent transgressive overyielding in this treatment is that the difference between the biomasses of the most productive and the average monoculture were small (14% compared to ~40% in the other treatments), which increases the probability that mixtures with non-transgressive overyielding also achieve transgressive overyielding [36]. The reason for the smaller yield differences among the monocultures in this treatment might be that carbon limitation through shading limited nutrient uptake, while in the mixtures complementarity in nutrient acquisition between different species was still large enough to cause transgressive overyielding.

### Do the effects of light and nutrient availability on diversity effects depend on functional group or growth stature composition?

In contrast to our expectations, we did not detect a dependency of trait-independent complementarity effects (TICE), trait-dependent complementarity effects (TDCE) or dominance effects (DE) on functional group or growth stature composition although the RYs of individual species varied dependent on functional group or growth stature identity at varying light availability (S2 Table). It has been shown in several biodiversity experiments that trait-independent complementarity effects depend on functional group (e.g. [2,13,31]) or growth stature composition [2]. Mostly, the effects of functional group composition were attributable to the inclusion of N_2_ fixing legumes, which are well known to facilitate the growth of neighbouring non-legumes. In our experiment, in particular the tall-statured grasses *A*. *elatius* and *D*. *glomerata* reached RY > 1, i.e. their performance in the mixtures was larger than expected from their monocultures. These species also reached the highest biomass production in absolute terms (S2 Fig). The same two tall-statured grass species have been shown previously to be highly productive both in monocultures and mixtures in the Jena Experiment [55,56] and other experimental grasslands [57]. Consistent with the resource-competition theory predicting the dominance of species with the greatest ability to efficiently acquire and use limiting resources [58], the superiority of these grass species in mixtures has been explained by their tall growth overtopping other species and their large amounts of biomass produced per unit nitrogen [59,60]. Although the RYs of the small-statured species were on average lower than the RYs of the tall-statured species, their biomass production in the mixtures was not lower than expected from their monocultures in case of small-statured grasses, while small-statured forb species underyielded. Consequently, the presence of the overyielding tall-statured grass species did not generally increase dominance effects, which would require that their biomass gain is at expense of other species. Obviously, the small-statured species included in our experimental species pool were to some degree able to compensate for greater canopy shade and reduced light supply in the presence of tall-statured species, while fertilization increased dominance effects irrespective of functional group or growth stature composition.

### Are there general relationships between diversity effects and functional trait composition?

Despite the lack of any relationships between TICE, TDCE or DE and the designed functional group or growth stature composition, we found significant relationships with functional trait composition in our exploratory analyses based on trait measurements in the field and weighted according to species biomass proportions in the mixtures. A smaller diversity in leaf nitrogen concentrations (FD_LNC_) was the most important predictor associated with larger TICE refuting our hypothesis of positive relationships between TICE and trait diversity. Leaf nitrogen concentrations are closely related to photosynthetic capacity [61]. A small diversity in LNC led to large TICE, when species showed similar values of LNC and thus had similar prerequisites for photosynthetic capacity and carbon assimilation. Our results are in contrast to findings from another biodiversity experiment (Jena Experiment) showing positive effects of FD_LNC_ on complementarity effects [22]. The Jena Experiment includes legumes, which had higher leaf nitrogen concentrations than grasses and forbs; thus facilitating effects of legumes caused the positive effects of FD_LNC_ on complementarity effects in the Jena Experiment. In addition, small community-weighted means of root nitrogen concentration (CWM_RNC_) and specific root length (CWM_SRL_) were important predictors related to large TICE. High root nitrogen concentrations and specific root length are indicators for high root respiration [14,62]. Root respiration is supposed to indicate a greater investment into root growth, nutrient uptake and transport, while reducing root carbon storage for the release of energy. Hence, mixtures with high CWM_RNC_ and CWM_SRL_ had supposedly higher root respiration rates and larger costs for nutrient uptake, which could explain the smaller extent of complementarity effects.

Interestingly, small FD_LNC_ were also the most important predictor for higher DE in our study. Low FD_LNC_ is not only possible if a mixture consists of species with similar leaf nitrogen concentrations, but low trait diversity could also be caused by the dominance of particular species. In addition to the negative relationships between DE and FD_LNC_, high community means in shoot length (high CMW_Hmax_) were associated with large DE in our experiment. Species which grow taller and produce more photosynthetically active tissue with high nitrogen concentrations, are likely to have a competitive advantage in light acquisition and may cause strong dominance effects [22,25,63]. Consequently, functional trait-based analysis implies that the dominance of tall-growing species (high CWM_Hmax_) with high leaf nitrogen concentrations (low FD_LNC_) most likely caused positive dominance effects, which were particularly large under fertilization. In contrast, TDCE were positive in the shade without fertilization (Fig 4c), while TDCE was close to zero in the other resource treatments. The chosen trait combination related to high TDCE indicated that tolerance of shading through increased SLA (high CWM_SLA_) under low-nutrient conditions (low CWM_LNC_) were important traits which caused a biomass gain of species with large monoculture biomass in the mixtures without negatively affecting other species.

## Conclusions

In summary, our experiment provided evidence that overyielding and the proportional biomass gain through diversity effects in mixtures of grass and forb species depends on the availability of soil resources and are more pronounced when nutrients are not available at excess. Our results strongly emphasize the need to incorporate both above- and belowground traits in trait-based approaches to identify the functional characteristics of the involved species, which are related to the varying extent of diversity effects and the relative importance of complementarity and dominance effects at different resource availability.

## Supporting Information

S1 Fig Effects of resource availability on species relative yields depending on (a) functional group identity, and (b) growth stature identity. (DOCX)

S2 Fig Species—level biomass production corrected by sown proportions averaged across all communities. (DOCX)

S1 File Description of functional trait measurements. (DOCX)

S1 Table Soil chemical properties of the experimental site. (DOCX)

S2 Table Summary of linear mixed-effects models for species relative yields (RYs) and species biomass corrected by sown proportions. (DOCX)

S3 Table Pearson correlation coefficients between trait-independent complementarity effects (TICE), trait-dependent complementarity effects (TDCE) and dominance effects (DE) and trait-based predictors based on community-weighted mean traits (CWM) and trait diversity (FD). (DOCX)

S4 Table Summary of coefficient estimates of the five best models from global models including all trait-based predictors and trait-independent complementarity effects (TICE), trait-dependent complementarity effects (TDCE) and dominance effects (DE) as response variables. (DOCX)

S5 Table Species and community biomass production in the study year 2013 based on the sum of two harvests (spring, summer). (DOCX)

S6 Table Relative yield totals (RYT), D_max_ and diversity effects derived from annual biomass production for each mixture. (DOCX)

S7 Table Community-weighted mean traits (CWM) and trait diversity (FD) for each mixture based on above- and belowground traits measured in each resource treatment and species biomass proportions in the mixtures. (DOCX)
