# Supplementary material for: Resource Availability Alters Biodiversity Effects in Experimental Grass-Forb Mixtures
Source: PLoS One. 2016 Jun 24;11(6):e0158110. doi: 10.1371/journal.pone.0158110 (PMC4920387; doi:10.1371/journal.pone.0158110)

**S1 Figure** Effects of resource availability on species relative yields dependent on (a) functional group identity, and (b) growth stature identity.

Shown are means (± 1 SE) across mixtures of different species richness for each resource treatment. The threshold for greater biomass production of a species in the mixtures than expected from its monoculture (RY > 1) is indicated with a dotted line. Abbreviations of treatments: F-S- = no fertilization, no shading, F-S+ = no fertilization, shading, F+S- = fertilization, no shading, and F+S+ = fertilization, shading.


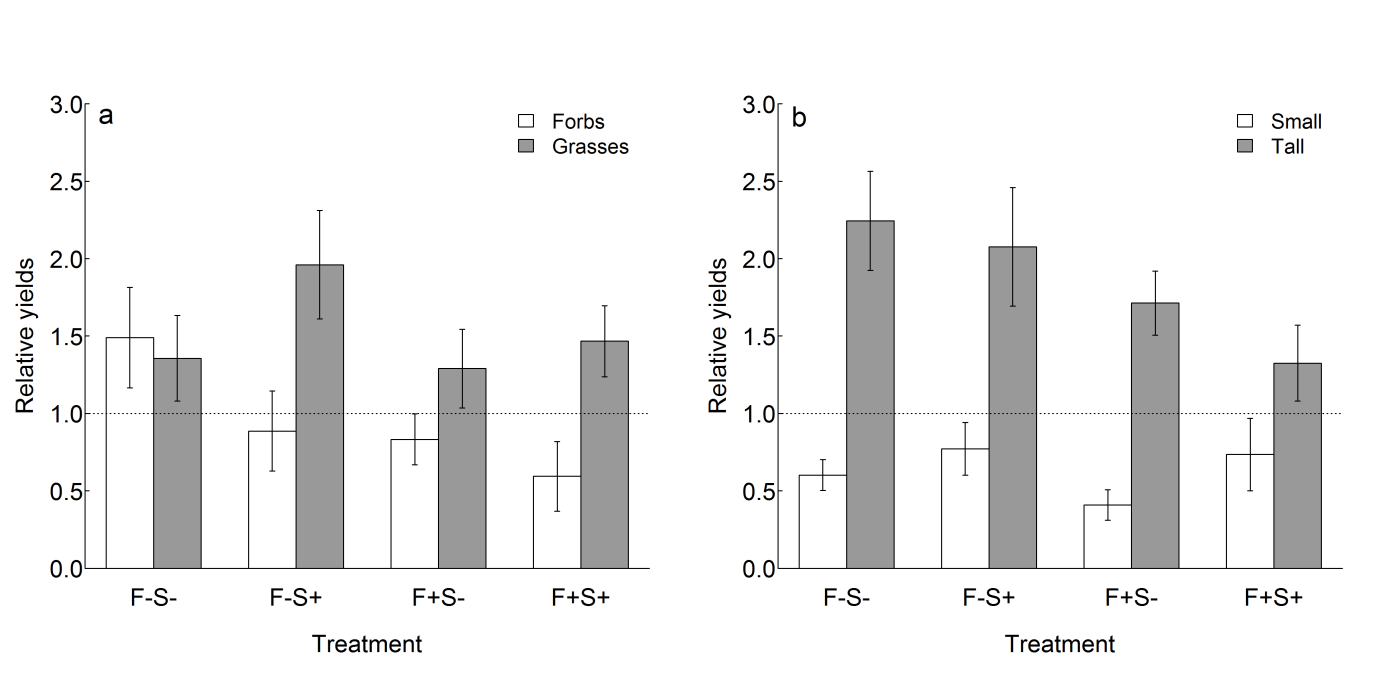

Supplement: S1 Fig — (DOCX) [file pone.0158110.s001.docx]
