# Supplementary material for: Resource Availability Alters Biodiversity Effects in Experimental Grass-Forb Mixtures
Source: PLoS One. 2016 Jun 24;11(6):e0158110. doi: 10.1371/journal.pone.0158110 (PMC4920387; doi:10.1371/journal.pone.0158110)
Supplement: S2 Fig — (DOCX) [file pone.0158110.s002.docx]

**S2 Figure** Species–level biomass corrected by sown proportions averaged across all communities.

Species biomass was multiplied by the number of sown species in the mixtures to account for decreasing sown proportions of individual species at increasing species richness. Shown are means (± 1 SE) across communities of different species richness and grown at varying resource availability. Different letters indicate significant differences among species in their biomass. Hatched bars = forbs, open bars = grasses, filled bars = small-statured species, unfilled bars = tall-statured species.

**
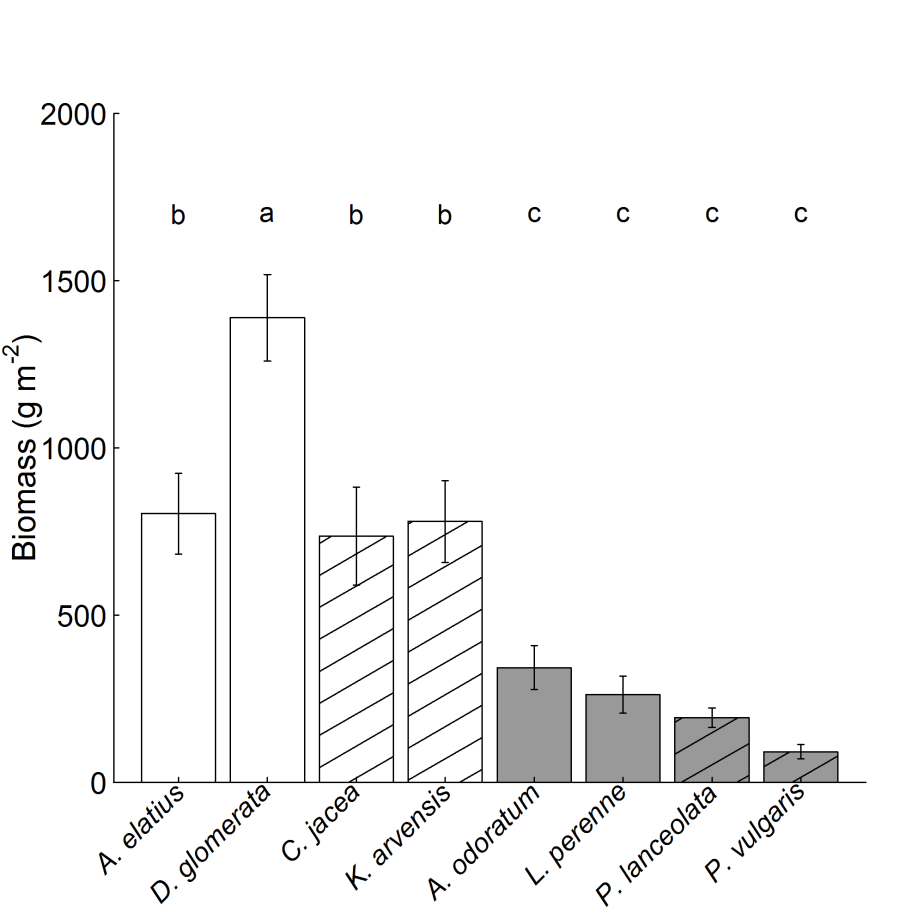
**
