# Supplementary material for: Resource Availability Alters Biodiversity Effects in Experimental Grass-Forb Mixtures
Source: PLoS One. 2016 Jun 24;11(6):e0158110. doi: 10.1371/journal.pone.0158110 (PMC4920387; doi:10.1371/journal.pone.0158110)
Supplement: S1 File — (DOCX) [file pone.0158110.s003.docx]

**S1 File: Functional trait measurements**

Shoots of each species were sampled for trait measurements in late May and August 2012, i.e. in the first year of treatment applications, in each plot. When only one life stage was available (vegetative or reproductive) five shoots per species were sampled, and when both life stages occurred four vegetative and four reproductive shoots were selected. Shoots were cut at ground level, and stored in a cooler for transport to the laboratory. There, stretched shoot length (H_max_; cm) was measured with a ruler. The area of three to five fully developed leaves (leaf blades in case of grasses) per shoot was determined with a leaf area meter (LI-3100 Area Meter, Li-COR, Lincoln, USA) and specific leaf area (SLA, leaf area per dry weight; mm^2^ mg^-1^) was calculated by dividing leaf area by leaf dry mass after drying at 70 °C for 48 h. Values of H_max_ and SLA obtained for each shoot were averaged per species and plot. Leaf samples pooled per species and plot (separately for each harvest) were ground to fine powder with a ball mill (Mixer Mill MM2000, Retsch, Haan, Germany). Leaf nitrogen concentrations (LNC; mg N g_leaf_^-1^) were measured with an elemental analyser (Vario EL Element Analyzer, Elementar, Hanau, Germany). Mean values of SLA, H_max_ and LNC across both harvests and plots with the same resource treatment were used for trait-based analyses.

Following first mowing in June 2013, i.e. in the second year of treatment applications, three soil cores per plot were sampled with a split-tube sampler (4.8 cm inner diameter; Eijkelkamp Agrisearch Equipment, Giesbeek, Netherlands) to a depth of 40 cm in each monoculture. Cores were taken with a minimum of 40 cm distance to the plot margin and between the samples within a plot. Each soil core was separated into 10 cm depth increments (0-10 cm, 10-20 cm, 20-30 cm and 30-40 cm) and layers were pooled per plot. Until further processing, samples were stored at -20° C. After thawing, samples were rinsed with tap water over a 0.5 mm sieve. Organic debris and remaining soil particles were removed with tweezers. Afterwards, root samples were scanned in a water filled tray on a flatbed scanner at 800 dpi. If sample volume exceeded tray capacity, subsamples were taken and kept separately. Dry mass of all samples was determined after drying at 70 °C for 48 h. The Winrhizo® Software (Regent Systems Inc., Quebec, Canada) was used to derive specific root length (SRL, root length per sample biomass; m g^-1^) from the images. SRL obtained per depth increment was weighted by root biomass of the respective layer to attain mean values over the depth profile. Weighted mean depth (WMD, cm) of vertical root biomass distribution was assessed as

$WMD= \frac{\sum_{i=1}^{S} MD* {BM}_{i}}{BM}$

where MD is the mean depth of a layer, BM_i_ is the root biomass of the respective layer and BM is total root biomass across all layers. Root samples of the different layers were pooled, ground to a fine powder and root nitrogen concentrations (RNC; mg N g_root_^-1^) were determined as described above.
