# Supplementary material for: Resource Availability Alters Biodiversity Effects in Experimental Grass-Forb Mixtures
Source: PLoS One. 2016 Jun 24;11(6):e0158110. doi: 10.1371/journal.pone.0158110 (PMC4920387; doi:10.1371/journal.pone.0158110)
Supplement: S1 Table — (DOCX) [file pone.0158110.s004.docx]

**S1 Table** Soil chemical properties of the experimental site

| Soil properties | Depth (cm) | Range | Mean (± SD) |
| --- | --- | --- | --- |
| Nitrogen concentration (mg g^-1^) | 0-15 | 2.03 – 2.35 | 2.18 (± 0.11) |
|  | 15-30 | 2.01 – 2.14 | 2.08 (± 0.05) |
| Organic carbon concentration (mg g^-1^) | 0-15 | 23.1 - 25.8 | 24.5 (± 0.8) |
|  | 15-30 | 22.6 - 23.8 | 23.3 (± 0.4) |
| CN ratio | 0-15 | 10.6 - 11.7 | 11.0 (± 0.3) |
|  | 15-30 | 10.6 - 11.1 | 10.9 (± 0.2) |
| Carbonate concentration (%) | 0-15 | 0.40 - 0.60 | 0.50 (± 0.06) |
|  | 15-30 | 0.47 - 0.70 | 0.60 (± 0.09) |
| pH | 0-15 | 6.68 - 7.22 | 6.93 (± 0.15) |
|  | 15-30 | 6.85 - 7.46 | 7.12 (± 0.20) |
| Phosphorus concentration (mg kg^-1^) | 0-15 | 36.7 - 42.3 | 39.3 (± 2.5) |
|  | 15-30 | 24.3 - 45.0 | 36.4 (± 7.2) |
| Potassium concentration (mg kg^-1^) | 0-15 | 102.0 - 247.0 | 151.7 (± 50.8) |
|  | 15-30 | 71.5 -168.0 | 107.1 (± 37.8) |

Soil samples were taken at three locations in each block (0-30 cm depth, separated into layers of 0-15 cm and 15-30 cm depth) and pooled block-wise before establishment of the experiment. Carbonate concentrations were determined according to Scheibler. Total carbon and nitrogen concentrations were measured with an elemental analyser (Vario EL Element Analyzer, Elementar, Hanau, Germany). Soil organic carbon concentrations were calculated by subtracting inorganic carbon concentrations from total carbon concentrations. The pH values were obtained after suspending the soil with 0.01M CaCl_2_. Phosphorus concentrations were measured from extracts with double lactate, potassium concentrations were obtained from calcium acetate lactate extracts. Shown are the range and means (± 1 SD) across blocks (N = 8).
