# Supplementary material for: Resource Availability Alters Biodiversity Effects in Experimental Grass-Forb Mixtures
Source: PLoS One. 2016 Jun 24;11(6):e0158110. doi: 10.1371/journal.pone.0158110 (PMC4920387; doi:10.1371/journal.pone.0158110)
Supplement: S2 Table — (DOCX) [file pone.0158110.s005.docx]

**S2 Table** Summary of linear mixed-effects models for species relative yields (RYs) and species-level biomass corrected by sown proportions

| Source of variation |  | Relative yields | | |  | Species biomass | | |  |
| --- | --- | --- | --- | --- | --- | --- | --- | --- | --- |
|  | df | MS | F | p |  | MS | F | p |  |
| Shade | 1 | 0.219 | 0.59 | 0.472 |  | 824875 | 5.39 | 0.062 |  |
| Fertilizer | 1 | **3.906** | **10.53** | **0.003** | **↓** | **1938696** | **12.67** | **0.001** | **↑** |
| Species richness (SR) | 1 | 0.034 | 0.09 | 0.769 |  | 152870 | 1.00 | 0.344 |  |
| Functional group identity (FG-ID) | 1 | **3.779** | **10.19** | **0.033** |  | 429821 | 2.81 | 0.173 |  |
| Growth stature identity (GS-ID) | 1 | **14.305** | **38.56** | **0.003** |  | **3517856** | **23.00** | **0.010** |  |
| Shade x Fertilizer | 1 | 0.066 | 0.18 | 0.675 |  | **1446948** | **9.46** | **0.003** |  |
| FG-ID x GS-ID | 1 | 1.183 | 3.19 | 0.148 |  | 103986 | 0.68 | 0.458 |  |
| Shade x SR | 1 | 0.516 | 1.39 | 0.250 |  | 3915 | 0.03 | 0.873 |  |
| Fertilizer x SR | 1 | 0.001 | <0.001 | 0.951 |  | 311203 | 2.03 | 0.156 |  |
| Shade x FG-ID | 1 | **6.066** | **16.35** | **<0.001** |  | **1354026** | **8.85** | **0.003** |  |
| Fertilizer x FG-ID | 1 | **0.676** | **1.82** | **0.180** |  | **1445873** | **9.45** | **0.003** |  |
| Shade x GS-ID | 1 | 1.980 | 5.34 | 0.023 |  | **1918993** | **12.55** | **0.001** |  |
| Fertilizer x GS-ID | 1 | 0.021 | 0.06 | 0.814 |  | **765562** | **5.01** | **0.027** |  |
| Shade x Fertilizer x SR | 1 | 0.111 | 0.30 | 0.589 |  | 107425 | 0.70 | 0.404 |  |
| Shade x Fertilizer x FG-ID | 1 | 0.101 | 0.27 | 0.603 |  | 412484 | 2.70 | 0.103 |  |
| Shade x Fertilizer x GS-ID | 1 | 0.101 | 0.27 | 0.603 |  | 857499 | **5.61** | **0.019** |  |

Species biomasses (BM_i_) and relative yields (RY_i_) were multiplied by the number of sown species in the mixtures prior to statistical analyses to account for decreasing sown proportions of individual species at increasing species richness. Approximate F statistics (type I SS using Kenward-Roger degrees of freedom approximation) was used to assess model improvement and the statistical significance of the fixed effects (p values). Significant effects are marked in bold. Arrows indicate an increase (↑) or decrease (↓) of the studied variable with shading or fertilizer addition. Note that analyses of species biomass included the monocultures, while relative yields refer to the mixtures.
