# Supplementary material for: Resource Availability Alters Biodiversity Effects in Experimental Grass-Forb Mixtures
Source: PLoS One. 2016 Jun 24;11(6):e0158110. doi: 10.1371/journal.pone.0158110 (PMC4920387; doi:10.1371/journal.pone.0158110)
Supplement: S3 Table — (DOCX) [file pone.0158110.s006.docx]

**S3 Table** Pearson correlation coefficients between trait-independent complementarity effects (TICE), trait-dependent complementarity effects (TDCE) and dominance effects (DE) and trait-based predictors based on community-weighted mean traits (CWM) and trait diversity (FD)

|  | TICE | | TDCE | | DE | |
| --- | --- | --- | --- | --- | --- | --- |
|  | r | p | r | p | r | p |
| Community-weighted mean traits | | |  |  |  |  |
| CWM_SLA_ | 0.032 | 0.806 | -0.191 | 0.137 | 0.059 | 0.651 |
| CWM_SRL_ | 0.017 | 0.894 | -0.201 | 0.118 | 0.055 | 0.674 |
| CWM_LNC_ | -0.107 | 0.406 | **-0.366** | **0.003** | -0.062 | 0.631 |
| CWM_RNC_ | -0.217 | 0.090 | -0.005 | 0.970 | 0.089 | 0.490 |
| CWM_Hmax_ | 0.128 | 0.320 | -0.020 | 0.875 | **0.327** | **0.010** |
| CWM_WMD_ | 0.029 | 0.826 | **0.294** | **0.020** | 0.117 | 0.366 |
| Trait diversity |  |  |  |  |  |  |
| FD_SLA_ | -0.214 | 0.095 | 0.210 | 0.102 | -0.118 | 0.360 |
| FD_SRL_ | -0.186 | 0.148 | 0.044 | 0.736 | **-0.252** | **0.048** |
| FD_LNC_ | **-0.285** | **0.025** | -0.009 | 0.942 | **-0.264** | **0.038** |
| FD_RNC_ | -0.139 | 0.281 | 0.052 | 0.686 | **-0.275** | **0.030** |
| FD_Hmax_ | -0.187 | 0.145 | <0.001 | 0.995 | -0.128 | 0.322 |
| FD_WMD_ | -0.105 | 0.418 | 0.075 | 0.561 | **-0.358** | **0.004** |

Note that correlation coefficients were calculated after accounting for block effects on TICE, TDCE and DE. Abbreviations for traits are: Hmax = shoot length, LNC = leaf nitrogen concentration, RNC = root nitrogen concentration, SLA = specific leaf area, SRL = specific root length, WMD = weighted mean depth of root biomass distribution.
