# Supplementary material for: Resource Availability Alters Biodiversity Effects in Experimental Grass-Forb Mixtures
Source: PLoS One. 2016 Jun 24;11(6):e0158110. doi: 10.1371/journal.pone.0158110 (PMC4920387; doi:10.1371/journal.pone.0158110)
Supplement: S4 Table — (DOCX) [file pone.0158110.s007.docx]

**S4 Table** Summary of coefficient estimates of the best models from global models including all trait-based predictors and trait-independent complementarity effects (TICE), trait-dependent complementarity effects (TDCE) and dominance effects (DE) as response variables

| Intercept | CWM_SLA_ | CWM_SRL_ | CWM_LNC_ | CWM_RNC_ | CWM_Hmax_ | CWM_WMD_ | FD_SLA_ | FD_SRL_ | FD_LNC_ | FD_RNC_ | FD_Hmax_ | FD_WMD_ | df | logLik | AIC | delta | weight |
| --- | --- | --- | --- | --- | --- | --- | --- | --- | --- | --- | --- | --- | --- | --- | --- | --- | --- |
| **Trait-independent complementarity effects (TICE)** | | | | | |  |  |  |  |  |  |  |  |  |  |  |  |
| 796.31 | - | -79.86 | - | -200.70 | - | - | - | - | -39.56 | - | - | - | 6 | -407.54 | 827.09 | 0.00 | 0.08 |
| 469.18 | - | - | - | -233.41 | - | - | - | - | -34.34 | - | - | - | 5 | -408.76 | 827.52 | 0.43 | 0.06 |
| 355.85 | - | - | - | -251.23 | - | 13.86 | - | - | -32.80 | - | - | - | 6 | -408.36 | 828.72 | 1.63 | 0.03 |
| 449.86 | - | - | - | -220.01 | - | - | - | - | -40.00 | - | - | 13.65 | 6 | -408.39 | 828.78 | 1.70 | 0.03 |
| 444.10 | - | -101.33 | - | - | - | - | - | - | -42.79 | - | - | - | 5 | -409.52 | 829.04 | 1.96 | 0.03 |
| **Trait-dependent complementarity effects (TDCE)** | | | | | |  | | | | | |  | | | | | |
| 331.62 | 6.30 | -23.26 | -107.87 | - | - | - | - | - | - | - | - | - | 6 | -302.96 | 617.93 | 0.00 | 0.11 |
| 121.35 | 4.16 | - | -82.33 | - | - | 4.67 | - | - | - | - | - | - | 6 | -304.06 | 620.12 | 2.19 | 0.04 |
| 219.69 | 4.38 | - | -97.74 | - | - | - | - | - | - | - | - | - | 5 | -305.24 | 620.48 | 2.55 | 0.03 |
| -66.68 | - | - | - | - | - | 6.02 | - | - | - | - | - | - | 4 | -306.55 | 621.09 | 3.16 | 0.02 |
| **Dominance effects (DE)** | | |  | | | | | |  | | | | | |  | | |
| -77.44 | - | - | - | - | 0.91 | - | - | - | -21.40 | - | - | - | 5 | -369.56 | 749.12 | 0.00 | 0.04 |
| -36.08 | - | - | - | - | - | - | - | - | -24.82 | - | - | - | 4 | -370.58 | 749.17 | 0.05 | 0.04 |
| -61.60 | - | - | - | - | 1.13 | - | 13.72 | - | -24.12 | - | - | - | 6 | -368.90 | 749.80 | 0.68 | 0.03 |
| 114.49 | - | - | -60.76 | - | 1.06 | - | - | - | -20.22 | - | - | - | 6 | -369.00 | 750.00 | 0.89 | 0.02 |
| -28.44 |  | - | - | - | - | - | - | - | -29.03 | - | 9.92 | - | 5 | -370.04 | 750.07 | 0.96 | 0.02 |

The best five models with delta <4 are shown if more models fulfilled this criterion and were included in model averaging. Block was entered as random effect. Models were automatically selected with the *R* package *MuMIn* [50] by comparing Akaike information criteria (AIC) as differences (delta) for models consisting of possible combinations of a maximum of three fixed effects. Abbreviations are: df = degrees of freedom, logLik = log likelihood, CWM = community-weighted mean traits, FD = trait diversity, Hmax = shoot length, LNC = leaf nitrogen concentration, RNC = root nitrogen concentration, SLA = specific leaf area, SRL = specific root length, WMD = weighted mean depth of root biomass distribution.
