# Supplementary material for: Resource Availability Alters Biodiversity Effects in Experimental Grass-Forb Mixtures
Source: PLoS One. 2016 Jun 24;11(6):e0158110. doi: 10.1371/journal.pone.0158110 (PMC4920387; doi:10.1371/journal.pone.0158110)
Supplement: S5 Table — (DOCX) [file pone.0158110.s008.docx]

**S5 Table** Species and community biomass production in the study year 2013 based on the sum of two harvests (spring, summer)

Plant communities of different species richness, functional group and growth stature composition were grown at different levels of resource availability manipulating light supply by shading and nutrient supply by fertilization. Each column gives the biomass of a species (g m^-2^), the last column shows community biomass (g m^-2^). NA indicates that a species did not belong to the sown species combination. Abbreviations are: Block = experimental block, Plot = experimental plot, Mix = mixture identity, S = shade (0 = control, 1 = shading), F = fertilization (0 = control, 1 = fertilization), SR = sown species richness, Pool = species pool of the mixture (see Table 1), FG = functional group composition (G = only grass species, F = only forb species, GF = grass and forb species), GS = growth stature composition (S = only small-statured species, T = only tall-statured species, ST = tall- and small-statured species), Ant_odo = biomass of *Anthoxanthum odoratum*, Arr_ela = biomass of *Arrhenatherum elatius*, Cen_jac = biomass of *Centaurea jacea*, Dac_glo = biomass of *Dactylis glomerata*, Kna_arv = biomass of *Knautia arvensis*, Lol_per = biomass of *Lolium perenne*, Pla_lan = biomass of *Plantago lanceolata*, Pru_vul = biomass of *Prunella vulgaris,* BM = community biomass (g m^-2^).

| Block | Plot | Mix | S | F | SR | Pool | FG | GS | Arr_ela | Cen_jac | Ant_odo | Pla_lan | Dac_glo | Kna_arv | Lol_per | Pru_vul | BM |
| --- | --- | --- | --- | --- | --- | --- | --- | --- | --- | --- | --- | --- | --- | --- | --- | --- | --- |
| B1 | A01 | S2M06 | 1 | 1 | 2 | A | GF | S | NA | NA | 576.7 | 5.0 | NA | NA | NA | NA | 581.7 |
| B1 | A02 | S2M03 | 1 | 0 | 2 | A | GF | ST | 197.2 | NA | NA | 117.1 | NA | NA | NA | NA | 314.2 |
| B1 | A03 | S2M04 | 1 | 0 | 2 | A | GF | ST | NA | 122.6 | 356.0 | NA | NA | NA | NA | NA | 478.6 |
| B1 | A04 | S2M07 | 1 | 0 | 2 | B | GF | T | NA | NA | NA | NA | 547.9 | 91.1 | NA | NA | 639.0 |
| B1 | A05 | S1M02 | 1 | 1 | 1 | A | F | T | NA | 332.7 | NA | NA | NA | NA | NA | NA | 332.7 |
| B1 | A06 | S1M01 | 1 | 0 | 1 | A | G | T | 395.5 | NA | NA | NA | NA | NA | NA | NA | 395.5 |
| B1 | A07 | S1M07 | 1 | 1 | 1 | B | G | S | NA | NA | NA | NA | NA | NA | 545.0 | NA | 545.0 |
| B1 | A08 | S4M01 | 1 | 1 | 4 | A | GF | ST | 281.4 | 0.0 | 210.5 | 6.3 | NA | NA | NA | NA | 498.2 |
| B1 | A09 | S1M08 | 1 | 0 | 1 | B | F | S | NA | NA | NA | NA | NA | NA | NA | 226.3 | 226.3 |
| B1 | A10 | S2M08 | 1 | 1 | 2 | B | GG | ST | NA | NA | NA | NA | 768.9 | NA | 0.0 | NA | 768.9 |
| B1 | A11 | S4M02 | 1 | 0 | 4 | B | GF | ST | NA | NA | NA | NA | 230.4 | 149.4 | 9.4 | 28.3 | 417.5 |
| B1 | A12 | S2M11 | 1 | 1 | 2 | B | FF | ST | NA | NA | NA | NA | NA | 393.9 | NA | 44.6 | 438.5 |
| B2 | A13 | S2M04 | 0 | 0 | 2 | A | GF | ST | NA | 269.3 | 57.4 | NA | NA | NA | NA | NA | 326.7 |
| B2 | A14 | S1M04 | 0 | 1 | 1 | A | F | S | NA | NA | NA | 504.8 | NA | NA | NA | NA | 504.8 |
| B2 | A15 | S2M11 | 0 | 1 | 2 | B | FF | ST | NA | NA | NA | NA | NA | 758.8 | NA | 80.8 | 839.5 |
| B2 | A16 | S2M12 | 0 | 0 | 2 | B | GF | S | NA | NA | NA | NA | NA | NA | 109.3 | 38.4 | 147.7 |
| B2 | A17 | S2M08 | 0 | 1 | 2 | B | GG | ST | NA | NA | NA | NA | 1214.7 | NA | 23.7 | NA | 1238.4 |
| B2 | A18 | S2M03 | 0 | 0 | 2 | A | GF | ST | 78.7 | NA | NA | 101.4 | NA | NA | NA | NA | 180.0 |
| B2 | A19 | S1M05 | 0 | 0 | 1 | B | G | T | NA | NA | NA | NA | 343.4 | NA | NA | NA | 343.4 |
| B2 | A20 | S4M02 | 0 | 0 | 4 | B | GF | ST | NA | NA | NA | NA | 393.7 | 17.5 | 1.8 | 13.5 | 426.5 |
| B2 | A21 | S4M01 | 0 | 1 | 4 | A | GF | ST | 298.7 | 237.9 | 39.8 | 62.3 | NA | NA | NA | NA | 638.6 |
| B2 | A22 | S1M03 | 0 | 0 | 1 | A | G | S | NA | NA | 481.3 | NA | NA | NA | NA | NA | 481.3 |
| B2 | A23 | S1M06 | 0 | 1 | 1 | B | F | T | NA | NA | NA | NA | NA | 776.6 | NA | NA | 776.6 |
| B2 | A24 | S2M01 | 0 | 1 | 2 | A | GF | T | 681.2 | 169.2 | NA | NA | NA | NA | NA | NA | 850.4 |
| B3 | A25 | S2M12 | 1 | 1 | 2 | B | GF | S | NA | NA | NA | NA | NA | NA | 508.7 | 0.5 | 509.2 |
| B3 | A26 | S2M11 | 1 | 0 | 2 | B | FF | ST | NA | NA | NA | NA | NA | 676.2 | NA | 2.5 | 678.7 |
| B3 | A27 | S2M01 | 1 | 0 | 2 | A | GF | T | 467.1 | 0.0 | NA | NA | NA | NA | NA | NA | 467.1 |
| B3 | A28 | S2M03 | 1 | 1 | 2 | A | GF | ST | 119.6 | NA | NA | 18.2 | NA | NA | NA | NA | 137.8 |
| B3 | A29 | S2M08 | 1 | 0 | 2 | B | GG | ST | NA | NA | NA | NA | 509.3 | NA | 2.9 | NA | 512.2 |
| B3 | A30 | S4M01 | 1 | 0 | 4 | A | GF | ST | 276.7 | 0.0 | 46.4 | 70.7 | NA | NA | NA | NA | 393.8 |
| B3 | A31 | S4M02 | 1 | 1 | 4 | B | GF | ST | NA | NA | NA | NA | 1038 | 1.1 | 0.0 | 0.0 | 1039.1 |
| B3 | A32 | S1M03 | 1 | 1 | 1 | A | G | S | NA | NA | 342.9 | NA | NA | NA | NA | NA | 342.9 |
| B3 | A33 | S2M04 | 1 | 1 | 2 | A | GF | ST | NA | 109.5 | 335.7 | NA | NA | NA | NA | NA | 445.2 |
| B3 | A34 | S1M06 | 1 | 0 | 1 | B | F | T | NA | NA | NA | NA | NA | 358.7 | NA | NA | 358.7 |
| B3 | A35 | S1M05 | 1 | 1 | 1 | B | G | T | NA | NA | NA | NA | 707.2 | NA | NA | NA | 707.2 |
| B3 | A36 | S1M04 | 1 | 0 | 1 | A | F | S | NA | NA | NA | 375.6 | NA | NA | NA | NA | 375.6 |
| B4 | A37 | S2M08 | 0 | 0 | 2 | B | GG | ST | NA | NA | NA | NA | 577.4 | NA | 35.4 | NA | 612.8 |
| B4 | A38 | S4M01 | 0 | 0 | 4 | A | GF | ST | 14 | 749.6 | 18.3 | 19.9 | NA | NA | NA | NA | 801.7 |
| B4 | A39 | S2M03 | 0 | 1 | 2 | A | GF | ST | 803.7 | NA | NA | 19.7 | NA | NA | NA | NA | 823.4 |

S5 Table continued:

| Block | Plot | Mix | S | F | SR | Pool | FG | GS | Arr_ela | Cen_jac | Ant_odo | Pla_lan | Dac_glo | Kna_arv | Lol_per | Pru_vul | BM |
| --- | --- | --- | --- | --- | --- | --- | --- | --- | --- | --- | --- | --- | --- | --- | --- | --- | --- |
| B4 | A40 | S2M06 | 0 | 0 | 2 | A | GF | S | NA | NA | 246.1 | 67.3 | NA | NA | NA | NA | 313.4 |
| B4 | A41 | S2M07 | 0 | 1 | 2 | B | GF | T | NA | NA | NA | NA | 914.5 | 80.5 | NA | NA | 995.0 |
| B4 | A42 | S1M08 | 0 | 1 | 1 | B | F | S | NA | NA | NA | NA | NA | NA | NA | 315.5 | 315.5 |
| B4 | A43 | S4M02 | 0 | 1 | 4 | B | GF | ST | NA | NA | NA | NA | 462.6 | 449.7 | 47.7 | 15.9 | 975.8 |
| B4 | A44 | S1M07 | 0 | 0 | 1 | B | G | S | NA | NA | NA | NA | NA | NA | 178.9 | NA | 178.9 |
| B4 | A45 | S1M02 | 0 | 0 | 1 | A | F | T | NA | 502.0 | NA | NA | NA | NA | NA | NA | 502.0 |
| B4 | A46 | S1M01 | 0 | 1 | 1 | A | G | T | 592.2 | NA | NA | NA | NA | NA | NA | NA | 592.2 |
| B4 | A47 | S2M11 | 0 | 0 | 2 | B | FF | ST | NA | NA | NA | NA | NA | 330.2 | NA | 24.7 | 354.9 |
| B4 | A48 | S2M04 | 0 | 1 | 2 | A | GF | ST | NA | 1008.4 | 125.8 | NA | NA | NA | NA | NA | 1134.2 |
| B5 | A49 | S1M08 | 1 | 1 | 1 | B | F | S | NA | NA | NA | NA | NA | NA | NA | 200 | 200.0 |
| B5 | A50 | S2M02 | 1 | 0 | 2 | A | GG | ST | 287.2 | NA | 53.4 | NA | NA | NA | NA | NA | 340.6 |
| B5 | A51 | S2M10 | 1 | 1 | 2 | B | GF | ST | NA | NA | NA | NA | NA | 250.6 | 216.5 | NA | 467.0 |
| B5 | A52 | S2M07 | 1 | 1 | 2 | B | GF | T | NA | NA | NA | NA | 834.3 | 15.5 | NA | NA | 849.8 |
| B5 | A53 | S2M09 | 1 | 1 | 2 | B | GF | ST | NA | NA | NA | NA | 777.8 | NA | NA | 1.3 | 779.1 |
| B5 | A54 | S2M05 | 1 | 0 | 2 | A | FF | ST | NA | 224.2 | NA | 172.7 | NA | NA | NA | NA | 396.8 |
| B5 | A55 | S4M02 | 1 | 0 | 4 | B | GF | ST | NA | NA | NA | NA | 379.4 | 147 | 8.4 | 7.8 | 542.6 |
| B5 | A56 | S1M02 | 1 | 0 | 1 | A | F | T | NA | 388.9 | NA | NA | NA | NA | NA | NA | 388.9 |
| B5 | A57 | S1M07 | 1 | 0 | 1 | B | G | S | NA | NA | NA | NA | NA | NA | 210.4 | NA | 210.4 |
| B5 | A58 | S2M06 | 1 | 0 | 2 | A | GF | S | NA | NA | 244.9 | 106.8 | NA | NA | NA | NA | 351.7 |
| B5 | A59 | S1M01 | 1 | 1 | 1 | A | G | T | 561 | NA | NA | NA | NA | NA | NA | NA | 561.0 |
| B5 | A60 | S4M01 | 1 | 1 | 4 | A | GF | ST | 363.9 | 0.1 | 19.2 | 18 | NA | NA | NA | NA | 401.3 |
| B6 | A61 | S2M02 | 0 | 0 | 2 | A | GG | ST | 274.1 | NA | 61.3 | NA | NA | NA | NA | NA | 335.4 |
| B6 | A62 | S4M02 | 0 | 0 | 4 | B | GF | ST | NA | NA | NA | NA | 168.9 | 384 | 26.5 | 6.8 | 586.3 |
| B6 | A63 | S1M04 | 0 | 0 | 1 | A | F | S | NA | NA | NA | 153.9 | NA | NA | NA | NA | 153.9 |
| B6 | A64 | S2M12 | 0 | 1 | 2 | B | GF | S | NA | NA | NA | NA | NA | NA | 397.9 | 208.9 | 606.7 |
| B6 | A65 | S2M05 | 0 | 0 | 2 | A | FF | ST | NA | 422.9 | NA | 117.4 | NA | NA | NA | NA | 540.3 |
| B6 | A66 | S2M09 | 0 | 1 | 2 | B | GF | ST | NA | NA | NA | NA | 1047.8 | NA | NA | 7.2 | 1055.0 |
| B6 | A67 | S1M03 | 0 | 1 | 1 | A | G | S | NA | NA | 670.6 | NA | NA | NA | NA | NA | 670.6 |
| B6 | A68 | S2M10 | 0 | 1 | 2 | B | GF | ST | NA | NA | NA | NA | NA | 1020.8 | 174.7 | NA | 1195.5 |
| B6 | A69 | S2M01 | 0 | 0 | 2 | A | GF | T | 293.0 | 578.6 | NA | NA | NA | NA | NA | NA | 871.6 |
| B6 | A70 | S4M01 | 0 | 1 | 4 | A | GF | ST | 352.6 | 435.2 | 2.2 | 40.5 | NA | NA | NA | NA | 830.5 |
| B6 | A71 | S1M06 | 0 | 0 | 1 | B | F | T | NA | NA | NA | NA | NA | 465.5 | NA | NA | 465.5 |
| B6 | A72 | S1M05 | 0 | 1 | 1 | B | G | T | NA | NA | NA | NA | 1326.7 | NA | NA | NA | 1326.7 |
| B7 | A73 | S2M09 | 1 | 0 | 2 | B | GF | ST | NA | NA | NA | NA | 711.9 | NA | NA | 19.2 | 731.1 |
| B7 | A74 | S2M02 | 1 | 1 | 2 | A | GG | ST | 221.0 | NA | 16.9 | NA | NA | NA | NA | NA | 237.8 |
| B7 | A75 | S4M02 | 1 | 1 | 4 | B | GF | ST | NA | NA | NA | NA | 493.2 | 9.6 | 0 | 3 | 505.7 |
| B7 | A76 | S2M10 | 1 | 0 | 2 | B | GF | ST | NA | NA | NA | NA | NA | 800.5 | 62.6 | NA | 863.1 |
| B7 | A77 | S1M03 | 1 | 0 | 1 | A | G | S | NA | NA | 333.2 | NA | NA | NA | NA | NA | 333.2 |
| B7 | A78 | S2M01 | 1 | 1 | 2 | A | GF | T | 491.1 | 6.1 | NA | NA | NA | NA | NA | NA | 497.2 |
| B7 | A79 | S1M04 | 1 | 1 | 1 | A | F | S | NA | NA | NA | 200.9 | NA | NA | NA | NA | 200.9 |
| B7 | A80 | S4M01 | 1 | 0 | 4 | A | GF | ST | 153.1 | 78.8 | 24.2 | 53.5 | NA | NA | NA | NA | 309.6 |
| B7 | A81 | S1M06 | 1 | 1 | 1 | B | F | T | NA | NA | NA | NA | NA | 1063.1 | NA | NA | 1063.1 |
| B7 | A82 | S2M12 | 1 | 0 | 2 | B | GF | S | NA | NA | NA | NA | NA | NA | 330.9 | 46.3 | 377.1 |
| B7 | A83 | S2M05 | 1 | 1 | 2 | A | FF | ST | NA | 41 | NA | 239.2 | NA | NA | NA | NA | 280.3 |
| B7 | A84 | S1M05 | 1 | 0 | 1 | B | G | T | NA | NA | NA | NA | 412 | NA | NA | NA | 412.0 |
| B8 | A85 | S1M08 | 0 | 0 | 1 | B | F | S | NA | NA | NA | NA | NA | NA | NA | 104.9 | 104.9 |
| B8 | A86 | S2M02 | 0 | 1 | 2 | A | GG | ST | 1279.5 | NA | 1.3 | NA | NA | NA | NA | NA | 1280.8 |
| B8 | A87 | S1M02 | 0 | 1 | 1 | A | F | T | NA | 1113.5 | NA | NA | NA | NA | NA | NA | 1113.5 |
| B8 | A88 | S1M07 | 0 | 1 | 1 | B | G | S | NA | NA | NA | NA | NA | NA | 551.4 | NA | 551.4 |
| B8 | A89 | S2M06 | 0 | 1 | 2 | A | GF | S | NA | NA | 387.3 | 172.1 | NA | NA | NA | NA | 559.4 |
| B8 | A90 | S2M09 | 0 | 0 | 2 | B | GF | ST | NA | NA | NA | NA | 356.4 | NA | NA | 3.2 | 359.6 |
| B8 | A91 | S2M10 | 0 | 0 | 2 | B | GF | ST | NA | NA | NA | NA | NA | 417.4 | 99.9 | NA | 517.3 |
| B8 | A92 | S1M01 | 0 | 0 | 1 | A | G | T | 184.9 | NA | NA | NA | NA | NA | NA | NA | 184.9 |
| B8 | A93 | S2M05 | 0 | 1 | 2 | A | FF | ST | NA | 427.6 | NA | 139.5 | NA | NA | NA | NA | 567.0 |
| B8 | A94 | S2M07 | 0 | 0 | 2 | B | GF | T | NA | NA | NA | NA | 308.6 | 113.5 | NA | NA | 422.1 |
| B8 | A95 | S4M02 | 0 | 1 | 4 | B | GF | ST | NA | NA | NA | NA | 493.8 | 493.9 | 31.4 | 26.7 | 1045.9 |
| B8 | A96 | S4M01 | 0 | 0 | 4 | A | GF | ST | 54.3 | 160.7 | 13.2 | 63.4 | NA | NA | NA | NA | 291.6 |
