# Supplementary material for: Resource Availability Alters Biodiversity Effects in Experimental Grass-Forb Mixtures
Source: PLoS One. 2016 Jun 24;11(6):e0158110. doi: 10.1371/journal.pone.0158110 (PMC4920387; doi:10.1371/journal.pone.0158110)
Supplement: S6 Table — (DOCX) [file pone.0158110.s009.docx]

**S6 Table** Relative yield totals (RYT), D_max_ and diversity effects derived from annual biomass production for each mixture

Plant communities of different species richness, functional group and growth stature composition were grown at different levels of resource availability manipulating light supply by shading and nutrient supply by fertilization. Abbreviations are: RYT = relative yield totals, D_max_ = transgressive overyielding, NE = net diversity effect (g m^-2^), TICE = trait-independent complementarity effect (g m^-2^), TDCE = trait-dependent complementarity effect (g m^-2^), DE = dominance effect (g m^-2^).

| Block | Plot | RYT | D_max_ | NE | TICE | TDCE | DE |
| --- | --- | --- | --- | --- | --- | --- | --- |
| B1 | A01 | 1.707 | 0.696 | 309.8 | 192.1 | 48.7 | 68.9 |
| B1 | A02 | 0.810 | -0.205 | -71.3 | -73.2 | -0.4 | 2.3 |
| B1 | A03 | 1.384 | 0.231 | 117.6 | 138.6 | -5.8 | -15.2 |
| B1 | A04 | 1.584 | 0.551 | 253.7 | 225.0 | 10.6 | 18.1 |
| B1 | A08 | 1.147 | -0.112 | 138.9 | 52.9 | 11.0 | 75.0 |
| B1 | A10 | 1.087 | 0.087 | 142.8 | 54.6 | 7.1 | 81.1 |
| B1 | A11 | 1.146 | 0.013 | 115.7 | 44.0 | 9.1 | 62.6 |
| B1 | A12 | 0.593 | -0.588 | -193.1 | -256.8 | -43.7 | 107.4 |
| B2 | A13 | 0.656 | -0.349 | -165.0 | -169.3 | -2.3 | 6.6 |
| B2 | A15 | 1.233 | 0.081 | 293.5 | 127.2 | 31.4 | 134.8 |
| B2 | A16 | 0.977 | -0.174 | 5.8 | -3.3 | -0.2 | 9.3 |
| B2 | A17 | 0.959 | -0.067 | 299.4 | -38.9 | -14.6 | 352.9 |
| B2 | A18 | 1.084 | -0.026 | 10.6 | 14.3 | -0.3 | -3.3 |
| B2 | A20 | 1.323 | -0.084 | 153.4 | 88.3 | 15.9 | 49.2 |
| B2 | A21 | 0.901 | -0.426 | -81.6 | -71.5 | 1.1 | -11.2 |
| B2 | A24 | 1.302 | -0.236 | -2.5 | 257.7 | -60.4 | -199.8 |
| B3 | A25 | 0.936 | -0.066 | 136.7 | -23.8 | -11 | 171.5 |
| B3 | A26 | 1.896 | 0.892 | 386.2 | 262.2 | 58.6 | 65.4 |
| B3 | A27 | 1.181 | 0.181 | 74.9 | 71.0 | 0.6 | 3.3 |
| B3 | A28 | 0.304 | -0.754 | -243.1 | -265.2 | -50.5 | 72.5 |
| B3 | A29 | 1.250 | 0.243 | 201.1 | 77.8 | 24.7 | 98.6 |
| B3 | A30 | 1.027 | -0.004 | 20.5 | 10.1 | 0.3 | 10.1 |
| B3 | A31 | 1.469 | -0.023 | 410.3 | 294.8 | 36.9 | 78.6 |
| B3 | A33 | 1.308 | 0.298 | 107.4 | 104.1 | 0.8 | 2.5 |
| B4 | A37 | 1.879 | 0.785 | 351.7 | 229.7 | 57.1 | 64.9 |
| B4 | A38 | 1.736 | 0.597 | 471.2 | 243.4 | 96.6 | 131.2 |
| B4 | A39 | 1.396 | 0.390 | 274.9 | 217.3 | 16.3 | 41.3 |
| B4 | A40 | 0.949 | -0.349 | -4.2 | -16.3 | -0.7 | 12.8 |
| B4 | A41 | 0.793 | -0.250 | -56.7 | -217.8 | -42.1 | 203.2 |
| B4 | A43 | 1.065 | -0.264 | 233.3 | 48.0 | 11.2 | 174.1 |
| B4 | A47 | 0.945 | -0.238 | 69.7 | -15.8 | -5.0 | 90.5 |
| B4 | A48 | 1.093 | 0.019 | 242.3 | 83.3 | 13.6 | 145.4 |
| B5 | A50 | 0.886 | -0.139 | -23.8 | -41.4 | -2.3 | 19.9 |
| B5 | A51 | 0.633 | -0.561 | -337 | -295.2 | 24.3 | -66.1 |
| B5 | A52 | 1.194 | -0.201 | -35.4 | 171.9 | -33.7 | -173.6 |
| B5 | A53 | 1.106 | 0.102 | 325.5 | 48.3 | 26.7 | 250.5 |
| B5 | A54 | 1.036 | 0.020 | 14.6 | 13.8 | 0.0 | 0.7 |
| B5 | A55 | 1.405 | 0.317 | 240.7 | 122.2 | 34.2 | 84.3 |
| B5 | A58 | 1.019 | -0.064 | -2.7 | 6.9 | -0.2 | -9.4 |
| B5 | A60 | 0.795 | -0.285 | 41.9 | -73.8 | -29.9 | 145.5 |
| B6 | A61 | 1.610 | -0.303 | 2.3 | 203.1 | -76 | -124.7 |
| B6 | A62 | 1.530 | 0.259 | 313.1 | 144.8 | 58.3 | 110.0 |
| B6 | A64 | 1.383 | 0.100 | 173.2 | 166.2 | 1.9 | 5.1 |
| B6 | A65 | 1.605 | 0.076 | 212.4 | 198.4 | 5.2 | 8.7 |
| B6 | A66 | 0.813 | -0.205 | 233.9 | -153.8 | -89.4 | 477.0 |
| B6 | A68 | 1.631 | 0.539 | 531.5 | 419.1 | 43.5 | 68.9 |
| B6 | A69 | 2.737 | 0.736 | 528.1 | 596.6 | -43.5 | -25.0 |
| B6 | A70 | 1.070 | -0.254 | 110.2 | 50.2 | 3.9 | 56.1 |
| B7 | A73 | 1.813 | 0.775 | 412.0 | 259.4 | 68.4 | 84.1 |

S6 Table continued:

| Block | Plot | RYT | D_max_ | NE | TICE | TDCE | DE |
| --- | --- | --- | --- | --- | --- | --- | --- |
| B7 | A74 | 0.443 | -0.576 | -214.1 | -251.7 | -47.2 | 84.8 |
| B7 | A75 | 0.721 | -0.524 | -123.1 | -175.2 | -20.1 | 72.3 |
| B7 | A76 | 2.530 | 1.406 | 578.6 | 435.2 | 86.7 | 56.7 |
| B7 | A78 | 0.894 | -0.114 | 50.3 | -47.5 | -11.6 | 109.5 |
| B7 | A80 | 0.805 | -0.217 | -63.7 | -72.8 | -2.2 | 11.4 |
| B7 | A82 | 1.777 | 0.666 | 158.8 | 169.7 | -4.8 | -6.1 |
| B7 | A83 | 1.314 | -0.158 | 13.5 | 83.8 | -16.8 | -53.5 |
| B8 | A86 | 2.163 | 0.91 | 649.5 | 734 | -45.5 | -39.1 |
| B8 | A89 | 0.919 | -0.166 | -28.3 | -47.9 | -1.7 | 21.3 |
| B8 | A90 | 1.068 | 0.047 | 135.5 | 15.3 | 7.7 | 112.4 |
| B8 | A91 | 1.455 | 0.111 | 195.1 | 146.6 | 15.2 | 33.3 |
| B8 | A93 | 0.660 | -0.491 | -242.1 | -274.9 | -16.9 | 49.7 |
| B8 | A94 | 1.142 | -0.093 | 17.6 | 57.6 | -5.0 | -35.0 |
| B8 | A95 | 1.150 | -0.212 | 303.3 | 111.3 | 25.0 | 167.0 |
| B8 | A96 | 1.053 | -0.419 | -38.9 | 17.6 | -2.8 | -53.6 |
