# Supplementary material for: Resource Availability Alters Biodiversity Effects in Experimental Grass-Forb Mixtures
Source: PLoS One. 2016 Jun 24;11(6):e0158110. doi: 10.1371/journal.pone.0158110 (PMC4920387; doi:10.1371/journal.pone.0158110)
Supplement: S7 Table — (DOCX) [file pone.0158110.s010.docx]

**S7 Table** Community-weighted mean traits (CWM) and trait diversity (FD) for each mixture based on above- and belowground traits measured in each resource treatment and species biomass proportions in the mixtures

Plant communities of different species richness, functional group and growth stature composition were grown at different levels of resource availability manipulating light supply by shading and nutrient supply by fertilization. Detailed information on trait measurements is given in S1 file. Abbreviations are: CWM = community-weighted mean traits, FD = trait diversity, Hmax = shoot length (cm), LNC = leaf nitrogen concentration (mg g^-1^), RNC = root nitrogen concentration (mg g^-1^), SLA = specific leaf area (mm^2^ mg^–1^), SRL = specific root length (m g^-1^), WMD = weighted mean depths of root biomass distribution (cm), NA = data not available due to a missing species in two-species mixtures.

| Block | Plot | CWM_SLA_ | CWM_SRL_ | CWM_LNC_ | CWM_RNC_ | CWM_Hmax_ | CWM_WMD_ | FD_SLA_ | FD_SRL_ | FD_LNC_ | FD_RNC_ | FD_Hmax_ | FD_WMD_ |
| --- | --- | --- | --- | --- | --- | --- | --- | --- | --- | --- | --- | --- | --- |
| B1 | A01 | 33.22 | 489.9 | 37.61 | 11.80 | 45.5 | 9.0 | 0.0184 | 0.0099 | 0.0144 | 0.0149 | 0.0097 | 0.0522 |
| B1 | A02 | 24.77 | 138.6 | 30.43 | 9.09 | 66.1 | 12.2 | 0.0169 | 0.1336 | 0.2771 | 0.9406 | 0.7387 | 0.0722 |
| B1 | A03 | 28.92 | 418.4 | 30.41 | 14.13 | 37.6 | 9.8 | 0.0706 | 1.4310 | 0.0461 | 0.0019 | 0.0066 | 1.0537 |
| B1 | A04 | 24.74 | 206.3 | 25.19 | 8.32 | 82.2 | 12.8 | 0.0037 | 0.2457 | 0.0140 | 0.2188 | 0.0001 | 0.0207 |
| B1 | A08 | 29.28 | 331.7 | 36.83 | 10.48 | 61.9 | 9.1 | 0.2625 | 0.6189 | 0.0251 | 0.1619 | 0.2715 | 0.0774 |
| B1 | A10 | 26.44 | 362.6 | 31.03 | 11.62 | 91.1 | 11.8 | NA | NA | NA | NA | NA | NA |
| B1 | A11 | 25.52 | 178.6 | 24.72 | 9.40 | 76.2 | 12.9 | 0.1396 | 0.4460 | 0.0417 | 0.3981 | 0.5937 | 0.1658 |
| B1 | A12 | 25.75 | 113.9 | 24.99 | 16.28 | 73.0 | 12.6 | 0.3121 | 0.3518 | 0.0606 | 0.0745 | 0.6538 | 0.0075 |
| B2 | A13 | 19.18 | 110.4 | 21.82 | 7.35 | 30.5 | 12.0 | 0.0862 | 1.7157 | 0.0768 | 0.0002 | 0.0083 | 0.8841 |
| B2 | A15 | 18.26 | 82.7 | 20.17 | 10.87 | 73.1 | 12.1 | 0.1846 | 0.1698 | 0.0318 | 0.3399 | 0.7197 | 0.0013 |
| B2 | A16 | 15.96 | 168.8 | 18.65 | 8.26 | 47.7 | 11.8 | 0.7272 | 0.2190 | 0.1205 | 0.0015 | 1.0968 | 0.0324 |
| B2 | A17 | 20.82 | 254.1 | 21.42 | 8.78 | 75.6 | 12.2 | 0.0474 | 0.0494 | 0.0016 | 0.0016 | 0.0587 | 0.0810 |
| B2 | A18 | 17.69 | 189.4 | 20.15 | 8.61 | 33.4 | 11.6 | 0.0901 | 0.0409 | 0.3778 | 1.0940 | 0.8124 | 0.0404 |
| B2 | A20 | 18.77 | 297.2 | 18.87 | 7.40 | 54.6 | 10.6 | 0.0204 | 0.2347 | 0.0148 | 0.0452 | 0.1457 | 0.1124 |
| B2 | A21 | 20.39 | 162.6 | 26.11 | 8.60 | 56.0 | 10.1 | 0.0613 | 0.6151 | 0.1423 | 0.4743 | 0.5901 | 0.4490 |
| B2 | A24 | 20.91 | 178.3 | 27.13 | 7.52 | 70.8 | 9.8 | 0.0077 | 0.3770 | 0.0080 | 0.3370 | 0.3257 | 0.2739 |
| B3 | A25 | 22.13 | 302.6 | 34.37 | 14.26 | 40.7 | 12.4 | 0.0055 | 0.0000 | 0.0006 | 0.0015 | 0.0019 | 0.0000 |
| B3 | A26 | 25.63 | 105.1 | 23.66 | 11.80 | 82.6 | 13.5 | 0.0059 | 0.0050 | 0.0000 | 0.0007 | 0.0317 | 0.0004 |
| B3 | A27 | 25.33 | 116.9 | 32.98 | 6.84 | 85.3 | 12.6 | NA | NA | NA | NA | NA | NA |
| B3 | A28 | 26.05 | 222.1 | 35.33 | 10.49 | 67.7 | 9.8 | 0.0234 | 0.0312 | 0.1481 | 0.4778 | 0.4876 | 0.6262 |
| B3 | A29 | 24.58 | 223.1 | 25.46 | 7.75 | 81.8 | 12.7 | 0.0024 | 0.0001 | 0.0016 | 0.0014 | 0.0184 | 0.0286 |
| B3 | A30 | 25.60 | 174.9 | 31.35 | 8.77 | 70.6 | 11.9 | 0.0744 | 0.8300 | 0.1782 | 0.9358 | 0.6028 | 0.4203 |
| B3 | A31 | 26.44 | 362.3 | 31.02 | 11.62 | 91.1 | 11.8 | 0.0001 | 0.0066 | 0.0013 | 0.0010 | 0.0001 | 0.0002 |
| B3 | A33 | 32.06 | 409.9 | 35.93 | 12.18 | 43.1 | 10.2 | 0.0968 | 0.8611 | 0.1782 | 0.0351 | 0.0311 | 1.2278 |
| B4 | A37 | 18.43 | 304.8 | 19.00 | 7.31 | 56.4 | 10.5 | 0.0906 | 0.0481 | 0.0007 | 0.0103 | 0.0025 | 0.0346 |
| B4 | A38 | 18.60 | 69.5 | 22.22 | 7.40 | 29.7 | 12.7 | 0.0196 | 0.4489 | 0.0396 | 0.0518 | 0.0352 | 0.1526 |
| B4 | A39 | 21.02 | 200.3 | 27.22 | 6.82 | 77.8 | 9.2 | 0.0164 | 0.0005 | 0.0407 | 0.0375 | 0.0868 | 0.0000 |
| B4 | A40 | 20.93 | 328.2 | 18.86 | 8.18 | 30.6 | 8.7 | 0.2659 | 0.1908 | 0.0204 | 0.2547 | 0.1539 | 0.4521 |
| B4 | A41 | 20.67 | 242.1 | 21.27 | 8.89 | 76.9 | 12.1 | 0.0435 | 0.3957 | 0.0084 | 0.0130 | 0.0008 | 0.0000 |
| B4 | A43 | 19.15 | 164.0 | 20.79 | 9.49 | 74.7 | 12.3 | 0.2254 | 1.2557 | 0.0387 | 0.1308 | 0.2762 | 0.2012 |
| B4 | A47 | 18.82 | 123.9 | 18.23 | 9.90 | 54.0 | 13.5 | 0.0297 | 0.0050 | 0.0178 | 0.0302 | 0.3206 | 0.1034 |
| B4 | A48 | 20.23 | 110.0 | 26.00 | 10.37 | 37.9 | 11.4 | 0.0084 | 0.4851 | 0.0027 | 0.0841 | 0.0006 | 0.3206 |
| B5 | A50 | 26.05 | 180.1 | 32.45 | 7.96 | 78.0 | 12.0 | 0.0705 | 1.0628 | 0.0344 | 0.6807 | 0.3242 | 0.5364 |
| B5 | A51 | 23.37 | 191.7 | 29.09 | 15.05 | 61.8 | 12.5 | 0.0483 | 1.1611 | 0.6191 | 0.0250 | 0.4435 | 0.0037 |
| B5 | A52 | 26.41 | 357.7 | 30.91 | 11.70 | 90.9 | 11.8 | 0.0021 | 0.1127 | 0.0217 | 0.0166 | 0.0018 | 0.0033 |
| B5 | A53 | 26.46 | 362.4 | 31.02 | 11.64 | 91.0 | 11.8 | 0.0039 | 0.0005 | 0.0001 | 0.0059 | 0.0153 | 0.0000 |
| B5 | A54 | 25.08 | 145.7 | 29.92 | 13.78 | 34.0 | 12.5 | 0.0375 | 0.1072 | 0.2814 | 0.0325 | 0.0002 | 0.1958 |
| B5 | A55 | 24.95 | 190.5 | 24.97 | 8.90 | 80.5 | 12.9 | 0.0411 | 0.3938 | 0.0303 | 0.3537 | 0.1678 | 0.1187 |
| B5 | A58 | 28.06 | 415.4 | 28.52 | 13.67 | 37.2 | 9.5 | 0.2111 | 0.9146 | 0.0707 | 0.0147 | 0.0094 | 0.4500 |
| B5 | A60 | 26.61 | 229.5 | 36.07 | 9.87 | 71.1 | 9.4 | 0.0584 | 0.1248 | 0.0576 | 0.1960 | 0.2188 | 0.2381 |
| B6 | A61 | 19.63 | 202.3 | 22.45 | 5.95 | 53.9 | 11.3 | 0.0632 | 0.3234 | 0.1188 | 0.1156 | 0.1109 | 0.6233 |
| B6 | A62 | 18.46 | 181.6 | 18.56 | 9.14 | 56.8 | 12.6 | 0.0761 | 0.6079 | 0.0107 | 0.2174 | 0.0585 | 0.5574 |
| B6 | A64 | 18.01 | 125.8 | 22.72 | 11.71 | 20.8 | 14.7 | 1.1741 | 0.1143 | 0.0001 | 1.6275 | 0.2274 | 1.0926 |
| B6 | A65 | 18.13 | 89.5 | 21.39 | 8.12 | 26.2 | 12.5 | 0.0399 | 0.9621 | 0.1968 | 0.2714 | 0.0873 | 0.1182 |
| B6 | A66 | 20.97 | 256.2 | 21.40 | 8.86 | 76.1 | 12.1 | 0.0033 | 0.0056 | 0.0005 | 0.0391 | 0.0524 | 0.0001 |

S7 Table continued:

| Block | Plot | CWM_SLA_ | CWM_SRL_ | CWM_LNC_ | CWM_RNC_ | CWM_Hmax_ | CWM_WMD_ | FD_SLA_ | FD_SRL_ | FD_LNC_ | FD_RNC_ | FD_Hmax_ | FD_WMD_ |
| --- | --- | --- | --- | --- | --- | --- | --- | --- | --- | --- | --- | --- | --- |
| B6 | A68 | 17.16 | 79.5 | 20.32 | 9.74 | 72.5 | 12.7 | 0.0846 | 0.0587 | 0.0487 | 0.0625 | 0.4377 | 0.5390 |
| B6 | A69 | 18.73 | 93.8 | 22.66 | 6.76 | 39.1 | 12.6 | 0.0033 | 0.8669 | 0.0060 | 0.1594 | 0.2704 | 0.0410 |
| B6 | A70 | 20.39 | 141.1 | 26.38 | 8.96 | 54.7 | 10.6 | 0.0352 | 0.5835 | 0.0784 | 0.5063 | 0.5402 | 0.4335 |
| B7 | A73 | 24.85 | 222.5 | 25.38 | 7.81 | 80.2 | 12.7 | 0.0529 | 0.0016 | 0.0051 | 0.0215 | 0.2159 | 0.0002 |
| B7 | A74 | 26.88 | 232.9 | 36.46 | 9.54 | 72.9 | 9.2 | 0.0689 | 0.1687 | 0.0018 | 0.0338 | 0.0649 | 0.0014 |
| B7 | A75 | 26.47 | 357.0 | 30.89 | 11.75 | 90.4 | 11.8 | 0.0157 | 0.1180 | 0.0228 | 0.0373 | 0.0544 | 0.0035 |
| B7 | A76 | 25.28 | 112.4 | 24.02 | 11.61 | 79.1 | 13.2 | 0.0460 | 0.1136 | 0.0520 | 0.0482 | 0.2262 | 0.4798 |
| B7 | A78 | 26.41 | 212.5 | 36.29 | 9.42 | 74.5 | 9.3 | 0.0011 | 0.0037 | 0.0080 | 0.0161 | 0.0238 | 0.0715 |
| B7 | A80 | 25.61 | 160.1 | 31.50 | 10.39 | 59.7 | 12.3 | 0.0563 | 0.5887 | 0.1711 | 1.2569 | 0.7525 | 0.3718 |
| B7 | A82 | 22.79 | 208.0 | 27.90 | 9.20 | 27.8 | 8.8 | 0.4700 | 0.0019 | 0.1048 | 0.0195 | 0.1286 | 0.5965 |
| B7 | A83 | 24.47 | 262.6 | 28.81 | 17.21 | 22.5 | 13.9 | 0.0705 | 0.1448 | 0.0130 | 0.0988 | 0.0544 | 0.0011 |
| B8 | A86 | 21.11 | 200.8 | 27.38 | 6.74 | 79.0 | 9.2 | 0.0000 | 0.0005 | 0.0002 | 0.0003 | 0.0019 | 0.0003 |
| B8 | A89 | 20.22 | 255.7 | 23.79 | 8.61 | 35.4 | 8.6 | 0.1758 | 0.1459 | 0.1842 | 0.1158 | 0.0704 | 0.0446 |
| B8 | A90 | 18.73 | 310.0 | 18.95 | 7.26 | 55.6 | 10.4 | 0.0038 | 0.0356 | 0.0041 | 0.0011 | 0.0418 | 0.0013 |
| B8 | A91 | 17.70 | 137.7 | 18.57 | 9.71 | 58.5 | 13.3 | 0.2497 | 0.0967 | 0.0111 | 0.0551 | 0.0040 | 0.1137 |
| B8 | A93 | 19.44 | 112.0 | 24.76 | 10.52 | 34.8 | 11.2 | 0.0707 | 0.3583 | 0.2231 | 0.0064 | 0.0460 | 0.3356 |
| B8 | A94 | 18.67 | 261.6 | 18.81 | 8.00 | 56.5 | 11.3 | 0.0001 | 0.5869 | 0.0046 | 0.2089 | 0.0005 | 0.5363 |
| B8 | A95 | 19.26 | 163.8 | 20.76 | 9.61 | 75.0 | 12.2 | 0.2113 | 1.2648 | 0.0372 | 0.1690 | 0.2898 | 0.1266 |
| B8 | A96 | 18.39 | 123.8 | 21.39 | 7.81 | 31.7 | 12.2 | 0.0784 | 1.4084 | 0.2247 | 0.4659 | 0.3360 | 0.3228 |
